# Smart Chemical Sensors for Monitoring and Detection of Spoilage in Fermented and Non-Fermented Food Products

**DOI:** 10.3390/s26165186

**Published:** 2026-08-16

**Authors:** Catarina Marques-Gomes, Fernanda Cosme, Ivo Oliveira, Berta Gonçalves, Teresa Pinto, António Inês, Alfredo Aires, Reinaldo Gomes, Sílvia Afonso, Alice Vilela

**Affiliations:** 1 Instituto Universitario de Investigación en Recursos Agrarios (INURA), Escuela de Ingenierías Industriales, Universidad de Extremadura, Avd. de la Investigación, 06006 Badajoz, Spain; catarinamg@unex.es; 2 Chemistry Research Center-Vila Real (CQ-VR), Department of Agronomy, School of Agrarian and Veterinary Sciences, University of Trás-os-Montes and Alto Douro, 5000 Vila Real, Portugal; 3 Chemistry Research Center-Vila Real (CQ-VR), Department of Biology and Environment, School of Life Sciences and Environment, University of Trás-os-Montes and Alto Douro, 5000-801 Vila Real, Portugal; fcosme@utad.pt (F.C.); aines@utad.pt (A.I.); 4 Center for the Research and Technology of Agro-Environmental and Biological Sciences (CITAB), Institute for Innovation, Capacity Building and Sustainability of Agri-Food Production (Inov4Agro), Department of Agronomy, School of Agrarian and Veterinary Sciences, University of Trás-os-Montes and Alto Douro, 5000-801 Vila Real, Portugal; ivo.vaz.oliveira@utad.pt (I.O.); alfredoa@utad.pt (A.A.); safonso@utad.pt (S.A.); 5 Center for the Research and Technology of Agro-Environmental and Biological Sciences (CITAB), Institute for Innovation, Capacity Building and Sustainability of Agri-Food Production (Inov4Agro), Department of Biology and Environment, School of Life Sciences and Environment, University of Trás-os-Montes and Alto Douro, 5000-801 Vila Real, Portugal; bertag@utad.pt (B.G.); tpinto@utad.pt (T.P.); 6 INESC TEC-Institute for Systems and Computer Engineering, Technology, and Science, Rua Dr. Roberto Frias, 4200-465 Porto, Portugal; reinaldo.luisgomes@gmail.com

**Keywords:** electrochemical sensors, food spoilage detection, chemiresistive sensors, microbial volatile organic compounds, intelligent packaging

## Abstract

Smart chemical sensors have emerged as promising tools for real-time monitoring of food spoilage in both fermented and non-fermented products. By detecting key spoilage indicators—including biogenic amines, ammonia, hydrogen sulfide, methane, pH variations, and microbial volatile organic compounds (MVOCs)—these systems enable rapid, on-site assessment of food quality, offering a viable alternative to conventional, time-consuming laboratory analyses. Recent advances encompass diverse sensing mechanisms, including chemiresistive platforms based on conducting polymers and MEMS (Microelectromechanical Systems); optical/colorimetric systems using dyes, metal–organic frameworks, and porphyrins; and electrochemical and biosensing approaches employing enzymes, antibodies, aptamers, and whole-cell recognition elements. These sensors demonstrate high sensitivity (ppb–ppm range), enabling early detection of spoilage before sensory perception or microbiological threshold exceedance. Their applicability has been validated across a wide range of food matrices, including meat, fish, dairy products, vegetables, beverages, and fermented foods. Despite significant progress, key challenges persist, including signal drift, limited specificity, susceptibility to environmental factors such as humidity and temperature, and interference from complex food matrices. Furthermore, integration into intelligent packaging requires the development of flexible, food-safe, and regulatory-compliant materials. Emerging approaches that combine sensor arrays with machine learning and MVOC pattern recognition are enhancing predictive accuracy and enabling food classification across commodity types. Overall, smart chemical sensing technologies are rapidly transitioning from laboratory prototypes to practical applications in intelligent packaging and wireless monitoring systems, with ongoing research focused on improving robustness, standardization, and scalability for commercial deployment. This article provides an overview of the topic, drawing on the available bibliography from the last five years and the most-cited scientific databases.

## 1. Introduction

### 1.1. Background on Food Spoilage Mechanisms

Usually, food spoilage mechanisms are divided into three types: physical, chemical, and microbial, which can sometimes overlap or influence one another. Physical spoilage refers to food spoilage caused by physical changes or instability, and the most common factors are moisture changes (loss, gain, or migration) and physical separation of components or ingredients [1,2]. Key factors affecting physical spoilage include moisture content, temperature, glass transition temperature, crystal growth, and crystallization. Food degradation often involves changes in moisture content, which may occur through water loss, gain, or migration [3]. Moisture transfer is directly related to water activity, defined as the ratio between the vapor pressure of water in the system and that of pure water at the same temperature [4]. Water activity decreases with temperature, which significantly influences food spoilage by affecting metabolic activity and ripening rates. Temperature is crucial for food stability, as low temperatures can cause freeze damage that may lead to cellular rupture. Slow or repeated freezing can worsen food degradation by promoting large ice crystals. Using emulsifiers and water-binding agents can limit this crystal growth. High sugar content increases the risk of sugar crystallization, leading to quality defects like staling in biscuits and graininess in candies and ice cream. This relates to the glass transition temperature, which affects texture and consumer acceptance [5]. Freezing can also degrade food, particularly when slow or repeated, because it forms large extracellular ice crystals. In high-sugar foods, sugar crystallization can occur due to moisture uptake or temperature increase, leading to undesirable visual and textural changes. However, adding fructose or starch can hinder it [5].

Food spoilage can also result from natural chemical and biochemical reactions that cause undesirable sensory changes [6]. This type of food spoilage involves oxidation, enzymatic reactions, and further chemical changes that deteriorate food quality [7]. Oxidation occurs in the presence of oxygen and can degrade amino acids, lipids, or pigments [1]. Proteases break down proteins into smaller peptides, and excessive activity can weaken protein structures, leading to undesirable texture changes and off-flavors [8,9]. Lipases hydrolyze lipids into fatty acids and glycerol, and their action is a primary cause of rancidity, unpleasant odors, and textural changes [10]. Amylases convert complex carbohydrates into simpler sugars such as glucose and fructose; in fruits and vegetables, this can result in starch breakdown, softer texture, and increased sweetness [11,12]. Oxidases catalyze oxidation reactions of food components, often causing changes in color and taste. For instance, polyphenol oxidase causes enzymatic browning in fruits and vegetables, producing undesirable discoloration [6,13]. Maillard reactions occur between amino groups in proteins and reducing sugars, resulting in darkening of color, reduced protein solubility, development of bitter flavor, and loss of nutritional quality [14].

Finally, another key factor for food spoilage is microbial growth. Microbial growth, primarily involving bacteria, yeasts, and molds, is a major contributor to food spoilage. Once established, they use food biopolymers, including proteins, polysaccharides, and lipids, as nutrient sources for growth and reproduction, making the food unacceptable to consumers [15]. These microorganisms typically contaminate food surfaces through exposure to soil, water, and air, and intrinsic factors such as endogenous enzymes and substrates, sensitivity to light, oxygen, pH, water activity, nutrient content, and oxidation–reduction potential influence their growth [16]. Extrinsic factors in food spoilage include relative humidity, temperature, and the presence and activity of other microbes [1]. Some microorganisms cause only food spoilage, while others can cause severe illness; others are even desirable for achieving the final properties of a given product (e.g., fermented foods). To control the microbial spoilage of foods, there are several options, including good industrial practices with strict attention to sanitation and hygiene, the adjustment of environmental conditions, like temperature, pH, water activity, or gas composition of the atmosphere, the use of preservation methods, including heat treatment, high-pressure processing, irradiation, or the use of antimicrobials [7,17,18].

### 1.2. Importance of Real-Time Monitoring in Food Systems

Real-time monitoring in food systems is increasingly recognized as a fundamental tool for ensuring food safety, quality, and operational efficiency across the entire supply chain [19]. By continuously tracking critical parameters such as temperature, humidity, pH, and gas composition, these systems provide valuable insights into the conditions that directly influence food stability and spoilage [20]. Maintaining appropriate environmental conditions is particularly important in cold chain processes, where minor temperature deviations can accelerate microbial growth, enzymatic reactions, and chemical degradation, ultimately compromising product quality and safety [21]. Advancements in sensing technologies, coupled with the integration of Internet of Things platforms, have significantly enhanced the ability to monitor food systems in real time [22]. Wireless sensors, smart labels, and connected devices enable continuous data collection and transmission, allowing stakeholders to respond promptly to any deviations from optimal storage or processing conditions. This real-time awareness is crucial for minimizing food losses, preventing spoilage, and ensuring that products meet quality standards before reaching consumers. Furthermore, such systems improve traceability by providing detailed records of environmental conditions throughout the supply chain, thereby increasing transparency and accountability [23]. The incorporation of advanced data analytics, machine learning, and artificial intelligence has further expanded the capabilities of real-time monitoring systems [24]. These technologies enable predictive modeling, allowing shelf-life estimation, identification of potential hazards, and optimization of logistics and storage strategies. For instance, predictive algorithms can analyze temperature fluctuations and microbial growth patterns to forecast spoilage, enabling proactive interventions rather than reactive measures [25]. This not only improves food safety but also helps reduce food waste, which remains a significant global challenge. In addition, real-time monitoring systems play a vital role in supporting food safety management frameworks, such as hazard analysis and critical control point (HACCP) systems [26]. Continuous data collection ensures that food products remain safe during processing, storage, and distribution. It enables early detection of contamination or quality issues, strengthening risk management and preventing foodborne illness. Real-time monitoring is especially important for perishable items like fruits and vegetables, where quality is sensitive to environmental changes. Sensor-based systems estimate the shelf life of fresh produce, offering more accurate predictions than traditional methods by monitoring parameters such as respiration rate and ethylene production, key indicators of ripening and spoilage [27].

### 1.3. Limitations of Conventional Detection Methods

Conventional detection methods for food spoilage have historically formed the backbone of food quality and safety assessment and remain the standards to follow [28,29]. These approaches primarily include microbiological, chemical, and sensory techniques widely used in laboratory settings [30]. Despite their extensive use, they present several limitations that restrict their effectiveness in modern food systems. One of the most common approaches is culture-based microbiological analysis, which relies on growing microorganisms on selective media to detect and enumerate them. However, it is inherently time-consuming, often requiring 24 to 72 h or more to produce reliable results [31], and it is also labor-intensive, requiring skilled personnel and controlled laboratory conditions. These requirements limit their application in rapid or on-site monitoring scenarios. Another major limitation is their inability to detect viable but non-culturable microorganisms that remain metabolically active yet fail to grow under standard laboratory conditions, leading to underestimation of microbial loads and posing a significant risk to food safety and quality [32]. Chemical analytical methods are also widely used to detect spoilage. Techniques such as gas chromatography and mass spectrometry identify volatile compounds associated with food deterioration [33], including those involved in meat spoilage [34]. While these techniques offer high sensitivity and specificity, they require sophisticated instrumentation and extensive sample preparation. This makes them expensive and less suitable for routine or field-based applications. Spectroscopic and metabolomic approaches have further enhanced the ability to detect spoilage-related chemical changes, but also depend on laboratory-based analysis and complex data interpretation [35]. Further methods, such as immunological and molecular techniques like enzyme-linked immunosorbent assays and polymerase chain reaction, offer targeted detection of specific microorganisms or toxins. Even though they provide higher sensitivity than conventional culture-based approaches [36], they still require specialized equipment and trained personnel. They can be limited by cross-reactivity and the need for sample preparation. Sensory evaluation remains a traditional method for assessing food spoilage, involving human assessment of attributes such as odor, taste, and appearance [37,38]. While it is directly relevant to consumer perception, it is inherently subjective and variable, as differences in individual sensitivity and experience can affect result reliability. Additionally, sensory methods cannot detect early spoilage, as noticeable changes typically occur only after significant chemical or microbial deterioration. Conventional detection methods are essential for food safety, but their limitations are becoming more apparent in today’s food systems. They require laboratory infrastructure, involve lengthy procedures, and lack real-time data, which limits their effectiveness. Innovative approaches are urgently needed to enable rapid, accurate, continuous monitoring. Integrating advanced technologies into food systems is crucial for improving spoilage detection and ensuring food quality. As research progresses, transitioning from conventional to smart detection systems will enhance food safety management and help reduce global food losses.

### 1.4. Emergence of Smart Chemical Sensors

Conventional methods are limited in their ability to integrate with modern digital food systems. With the increasing demand for real-time data and automation, traditional approaches are often inadequate because they cannot provide the continuous data flows necessary for predictive modeling and process optimization [39]. This gap has driven the development of advanced sensing technologies and smart monitoring systems [40] that can continuously detect chemical and biochemical changes associated with food deterioration. These can enable rapid, non-destructive, and highly sensitive monitoring of spoilage markers directly within food packaging or storage environments, operating via diverse methods, including detection of volatile organic compounds, gases, metabolites, or pH variations generated during microbial growth and enzymatic degradation [41]. For instance, compounds such as ammonia, hydrogen sulfide, carbon dioxide, and trimethylamine are commonly released during the spoilage of meat, seafood, and dairy products [42]. Advanced sensing materials, including graphene, carbon nanotubes, metal nanoparticles, and conductive polymers, have substantially improved sensor sensitivity, selectivity, and response time [43]. When integrated into colorimetric and electrochemical sensors, they provide visual or electrical responses that are easy to interpret for freshness evaluation. Integrating smart sensors with wireless communication technologies, such as RFID, NFC, and Internet of Things (IoT) platforms, has further enhanced food monitoring systems by enabling real-time data transmission and remote quality tracking throughout the supply chain [44,45]. In addition, electronic nose systems, combined with machine learning algorithms, can analyze complex odor patterns to predict spoilage stages and accurately estimate product shelf life [46,47,48]. These intelligent sensing systems help reduce food waste, improve food safety, and support sustainable food management practices. Despite considerable progress, challenges related to sensor stability, environmental interference, large-scale commercialization, and nanomaterial safety still require further investigation [49].

While these intelligent sensing systems offer significant advances in real-time food quality monitoring and spoilage prediction, their implementation also aligns with the growing emphasis on food safety and traceability across the supply chain. In the European Union, food traceability has become a legal requirement [50], reinforcing the need for reliable monitoring technologies that can continuously track product quality and support informed decision-making throughout distribution and storage. As a result, the food industry, retailers, consumers, and other stakeholders are increasingly interested in developing devices that are simple, low-cost, rapid, reliable, non-invasive, and non-destructive for real-time assessment of food freshness. One promising approach to meet these requirements is smart packaging technologies, particularly food spoilage indicators that monitor freshness directly within the package. These indicators typically detect chemical changes associated with microbial growth, offering a practical alternative to conventional sensory and microbiological analyses, which are often time-consuming, labor-intensive, and costly. By enabling continuous, real-time monitoring, such systems can significantly improve food quality control and safety management [51,52].

Because psychrotrophic microorganisms largely drive microbial spoilage under refrigerated conditions, metabolites and gases such as ammonia, trimethylamine, hydrogen sulfide, and carbon dioxide have emerged as valuable indicators of food quality deterioration [53,54,55,56]. These compounds are therefore widely explored as target markers in smart packaging and sensing technologies for real-time freshness monitoring [55,56,57].

Beyond gaseous metabolites, microbial activity also produces a wide range of volatile organic compounds (VOCs) and biogenic amines closely associated with food deterioration that can serve as early indicators of spoilage in both plant- and animal-based products [58,59,60,61,62,63,64,65,66]. Owing to their strong correlation with microbial growth and quality loss, these compounds have attracted considerable attention as target analytes in smart sensing and intelligent packaging systems for real-time, non-destructive food quality assessment [67,68,69].

Table 1 summarizes the main spoilage-related chemical markers relevant to smart sensing technologies.

## 2. Smart Chemical Sensors: Concepts and Classification

Smart chemical sensors are an emerging class of analytical devices that detect specific chemicals or physicochemical changes and convert them into quantifiable signals [71]. Unlike conventional sensing platforms, smart sensors incorporate advanced functionalities, such as embedded data processing, enabling autonomous operation and improving the reliability, accessibility, and interpretability of analytical outputs [72].

In recent years, smart chemical sensors have become essential tools for real-time food quality assessment. Their ability to detect, process, and transmit chemical information supports rapid decision-making across the food supply chain [73]. Within food monitoring applications, chemical sensors are fundamental to continuously measuring spoilage markers in the product’s headspace or surrounding environment [74]. Smart sensors extend this functionality by integrating onboard computation and remote connectivity, thereby improving data handling, enabling predictive analytics [75,76], and thus facilitating continuous monitoring.

The performance of smart chemical sensors is primarily determined by their sensitivity [77,78], selectivity [79,80,81,82,83,84,85], and response time [86,87], which collectively govern the accurate, rapid, and reliable detection of food spoilage markers under real-world monitoring conditions.

Chemical sensors include several major classes, each relying on distinct transduction mechanisms and offering complementary advantages for detecting spoilage-related markers. The most widely applied categories include electrochemical sensors [88,89,90], optical sensors [91,92], fluorescence sensors [93,94], gas sensors [95,96,97], and biosensors [98,99], all of which have shown significant progress in sensitivity, selectivity, and integration with smart monitoring platforms.

Beyond their distinct sensing mechanisms, modern smart chemical sensors increasingly incorporate advanced functionalities such as IoT connectivity, secure data transmission, and miniaturized designs, enabling real-time monitoring, remote data access, and improved traceability throughout the food supply chain [100,101,102,103]. Furthermore, advances in microfabrication, flexible electronics, and low-power sensor technologies have enabled compact, portable sensing platforms suitable for integration into food packaging and on-site quality assessment [104,105,106].

To facilitate comparison among the main sensing approaches, Table 2 summarizes representative examples reported in the literature for different spoilage markers and food matrices.

## 3. Materials and Nanotechnology in Sensor Development

The performance of food spoilage sensors is strongly influenced by the materials used in the sensing layer. To improve sensitivity, selectivity, and operational stability, researchers have incorporated a wide range of materials into sensing platforms designed for food quality monitoring [40,41]. These materials contribute differently to sensor performance, depending on the target analyte and sensing mechanism employed [124,125,126].

Among them, metal nanoparticles have attracted considerable attention because of their ability to enhance signal transduction and improve analytical sensitivity. Gold nanoparticles (AuNPs), for example, are frequently incorporated into optical and electrochemical sensors because of their plasmonic properties and signal-amplification capabilities [127,128,129]. In food-quality applications, AuNP-based sensors have been successfully employed to detect biogenic amines, including histamine, tyramine, and cadaverine [130,131,132,133,134,135,136,137]. Likewise, silver nanoparticles (AgNPs) have been integrated into sensing systems to monitor volatile spoilage compounds generated during the deterioration of meat, seafood, dairy products, fruits, and vegetables [138,139,140,141,142,143]. AgNPs have additionally been used for colorimetric detection of melamine in milk, monitoring postharvest spoilage in bananas and onions, and detecting biogenic amines such as trimethylamine using hydrogel-based sensing systems [144,145,146].

Developing environmentally friendly synthesis strategies alongside harmonized regulatory frameworks will also be essential for the safe commercialization and widespread adoption of intelligent nanoparticle-enabled food packaging [147].

In addition to metal nanoparticles, metal-oxide semiconductors (MOS) are among the most widely used sensing materials for monitoring food spoilage. Materials such as SnO_2_, ZnO, TiO_2_, and WO_3_ exhibit high responsiveness towards volatile compounds released during food deterioration [148,149]. Consequently, they constitute the basis of numerous gas sensors and electronic nose systems used for freshness assessment in meat, fish, dairy products, fruits, and vegetables [107,150,151,152,153,154].

Similarly, carbon-based nanomaterials have emerged as promising alternatives for food-quality sensing applications [155,156]. Graphene and carbon nanotubes (CNTs) are particularly attractive due to their high electrical conductivity and large surface area [157,158,159]. These characteristics have facilitated their incorporation into chemiresistive and electrochemical sensors, where they enable sensitive detection of spoilage-related compounds and support the development of intelligent packaging technologies [160,161,162,163].

Beyond nanostructured inorganic materials, functional polymers have also played a significant role in developing food spoilage sensors [164]. These materials offer several advantages, including flexibility, low cost, lightweight structure, chemical tunability, and compatibility with intelligent packaging technologies [165,166]. Conductive polymers such as polyaniline and polypyrrole have been widely explored for gas-sensing applications because they respond to chemical changes in the surrounding environment [167].

Conductive polymers such as polyaniline (PANI), polypyrrole (PPy), and polythiophene are particularly attractive for electrochemical and chemiresistive sensing applications because of their excellent electrical conductivity and environmental stability [111]. These polymers exhibit measurable changes in conductivity upon exposure to spoilage gases, enabling rapid detection of food freshness deterioration. Polymer composites containing nanoparticles, metal oxides, carbon nanomaterials, or natural dyes have demonstrated enhanced sensing performance due to synergistic effects between the polymer matrix and functional additives [168,169].

Moreover, researchers have extensively investigated polymeric films containing natural colorimetric indicators, including anthocyanins and curcumin, as freshness indicators for meat, fish, and seafood products [170,171]. Their compatibility with packaging materials makes them particularly attractive for intelligent packaging applications [164,167].

Recent advances have further expanded the applicability of polymer-based sensing materials through the development of hydrogel sensors [172,173,174], molecularly imprinted polymers [149], and digitally connected sensing platforms [175,176], enabling more selective detection of spoilage-related compounds and supporting real-time food monitoring applications. Nevertheless, long-term stability, environmental robustness, reproducibility, and large-scale commercialization continue to limit broader implementation [177].

While the materials discussed above primarily enhance signal generation and transduction, incorporating biological or biomimetic recognition elements can improve analytical selectivity [178,179,180,181]. Enzymes are frequently employed in biosensors because of their catalytic specificity [182,183], whereas antibodies provide selective recognition of target compounds and microorganisms [184,185]. In parallel, molecularly imprinted polymers (MIPs) have emerged as robust synthetic alternatives for the selective detection of compounds such as histamine and tyramine [186,187,188]. This approach has further expanded selective food sensing by enabling stable, highly specific recognition of spoilage-related compounds and other food contaminants [189,190,191]. Nevertheless, limitations in long-term performance and reproducibility remain important challenges to the broader application of biorecognition-based sensors [192].

## 4. Sensor Mechanisms for Spoilage Detection

### 4.1. Detection of Volatile Compounds

Volatile organic compounds (VOCs) are among the earliest and most informative chemical indicators of food spoilage. Microbial metabolism, enzymatic activity, and oxidation generate alcohols, aldehydes, ketones, esters, organic acids, sulfur-containing compounds, and amines that contribute to characteristic spoilage odors [112,122]. Because many of these compounds are released before visible deterioration or sensory rejection occurs, VOC monitoring has become one of the most widely used approaches for rapid food-quality assessment.

VOC composition varies with food type, storage conditions, and dominant spoilage microorganisms. In meat and seafood products, nitrogen- and sulfur-containing compounds are frequently associated with microbial degradation, whereas fruits and vegetables typically exhibit changes in aldehydes, alcohols, esters, and other metabolites associated with ripening and senescence [58,121]. As a result, VOC profiles can provide characteristic chemical fingerprints that reflect freshness status and the progression of spoilage.

Several sensing technologies have been developed for VOC monitoring, including gas sensors, optical sensors, and electronic nose systems [95,122,193]. Among these, electronic noses have attracted particular attention because they can analyze complex mixtures of volatile compounds rather than targeting a single analyte [69,121]. By combining sensor arrays with chemometric and machine-learning tools, these systems can distinguish freshness levels, identify spoilage stages, and support shelf-life estimation across a wide range of food products [48,122].

Recent studies have demonstrated the successful application of VOC-based sensing approaches in meat and fish products [69], dairy and fermented foods, and fruits and vegetables [27,121]. Their non-destructive nature and potential for real-time monitoring make them particularly attractive for intelligent packaging and food quality management systems [30,41]. Continued advances in sensor materials, miniaturization, and data analysis are expected to further improve the reliability and practical implementation of VOC-based spoilage monitoring technologies [43,122]. Incorporating advanced materials such as graphene, carbon nanotubes, and conducting polymers, as well as hybrid sensor systems, has further enhanced the performance of these sensing platforms, particularly for VOC monitoring applications [194,195,196]. Furthermore, integrating these sensors with established analytical techniques has improved calibration and reliability for food-quality assessment [197].

### 4.2. Detection of pH Changes

Sensor mechanisms for food spoilage detection primarily rely on monitoring physicochemical changes associated with microbial growth and metabolic activity, particularly pH variations (Table 3). During spoilage, bacterial protein breakdown produces alkaline compounds that raise pH, whereas glucose fermentation produces organic acids, such as acetic acid, lowering pH [198]. Consequently, pH is a crucial indicator of food freshness and the extent of spoilage [199].

These pH variations provide a reliable basis for freshness assessment and are widely exploited in colorimetric sensing systems. Common approaches include sensors based on pH-sensitive natural dyes, such as anthocyanins and curcumin. The color changes in these dyes are attributed to phenolic or conjugated structures that undergo structural transformations in response to pH changes [200]. As a result, these compounds show distinct, visible color shifts, providing a simple, rapid, and effective way to indicate spoilage. Consequently, colorimetric pH-responsive systems play a critical role in maintaining food quality and safety standards.

Several studies have demonstrated the practical application of such systems. For example, Silva-Pereira et al. [117] developed a pH indicator for monitoring the deterioration of fresh fish fillets using a film composed of chitosan, corn starch, and red cabbage extract. The indicator showed optical and morphological changes sensitive to pH during storage, highlighting its potential as an effective quality indicator.

Such sensing technologies are frequently integrated into smart packaging systems, allowing real-time, non-destructive monitoring of food freshness. More recently, integrating artificial intelligence (AI) into intelligent packaging has further enhanced the performance of these systems. By analyzing complex data patterns, AI-driven models improve the accuracy and sensitivity of spoilage detection and enable real-time prediction of changes in food quality [57].

Recent advances highlight the use of convolutional neural networks (CNNs) and deep learning (DL) techniques to interpret colorimetric sensor data, achieving classification accuracies exceeding 95% [201,202]. Hybrid approaches that combine traditional statistical methods, such as Random Forest (RF) and Support Vector Machine (SVM), with AI-based analytics have also improved performance. These integrated systems enhance the precision and robustness of spoilage detection and can be implemented on smartphone-based platforms, enabling portable, user-friendly monitoring. Notably, these applications can identify subtle or otherwise undetectable signs of spoilage [203].

For example, Gong et al. [204] developed a smartphone-based meat freshness monitoring system in which a hydrogel strip containing the pH-sensitive dye bromocresol green was embedded within the packaging. The strip responded to volatile amines produced during spoilage, generating a visible color change that a CNN-based smartphone application then analyzed to automatically determine meat freshness. In another study, a color-shifting silver nanoparticle sensor combined with smartphone imaging and an artificial neural network (ANN) detected chicken spoilage. These examples further demonstrate how AI can enhance the sensitivity and analytical capability of simple colorimetric indicators in intelligent packaging systems [204].

**Table 3:** Sensor mechanisms based on pH changes for spoilage detection.

Sensor Mechanism	Principle of Detection	pH Indicator System	Main Application	Advantages	Limitations	References
Colorimetric pH sensors	Visual color change due to pH variation from microbial activity	Natural dyes [anthocyanins, curcumin, bromocresol green]	Meat, fish, and dairy packaging	Simple, low-cost, real-time monitoring	Semi-quantitative, affected by light/storage	[205]
pH indicator films/coatings	Polymer films change color with pH variation	Biopolymer films with anthocyanins or curcumin	Packaging labels and coatings	Easy integration, biodegradable	Moisture sensitivity	[206]
Smart label pH sensors	Color change triggered by volatile basic/acidic compounds	Anthocyanin-based labels	Real-time freshness indicators	User-friendly, non-destructive	Environmental interference	[116,207]

### 4.3. Detection of Microbial Metabolites

Detecting microbial metabolites is one of the most effective approaches for monitoring food spoilage, as microbial growth in food systems produces specific chemical compounds that reflect both the type and extent of degradation. Microorganisms, including bacteria, yeasts, and molds, primarily drive spoilage by enzymatically breaking down proteins, lipids, and carbohydrates. As a result, a wide range of metabolites are produced, including biogenic amines, organic acids, volatile organic compounds (VOCs), and sulfur- and nitrogen-containing gases. The presence, concentration, and evolution of these compounds over time provide reliable indicators of food freshness and safety. Among these metabolites, VOCs are particularly significant because they are directly associated with sensory changes in food. VOCs include esters, alcohols, ketones, aldehydes, and sulfur-containing compounds, all of which contribute to the characteristic odors of spoiled food and strongly indicate microbial activity [58]. Of special importance is hydrogen sulfide (H_2_S), a sulfur-containing gas produced during the degradation of sulfur-containing amino acids. Its presence is closely linked to advanced stages of protein decomposition, making it a valuable spoilage marker [57].

Closely related to protein degradation are biogenic amines, such as histamine, tyramine, putrescine, and cadaverine. Spoilage bacteria produce these compounds by decarboxylating amino acids and are strongly associated with protein-rich foods such as fish and meat. Because of their toxicological relevance and strong correlation with microbial activity, researchers commonly detect them using enzymatic biosensors (e.g., amine oxidase-based systems) and electrochemical sensors [61,62].

Another important group of metabolites includes volatile nitrogen and sulfur compounds, particularly ammonia (NH_3_), hydrogen sulfide (H_2_S), and trimethylamine (TMA). Microbial decomposition of amino acids and other nitrogenous compounds generates these compounds. They are widely recognized as key spoilage markers in meat and fish because they form early and have strong odors. Gas sensors, such as metal-oxide-semiconductor (MOS) sensors, typically detect them by measuring changes in electrical properties [208] (Table 4).

In addition to protein-derived metabolites, organic acids, such as lactic, acetic, and butyric acids, are produced through microbial fermentation of carbohydrates. These compounds are particularly relevant in dairy products and plant-based foods, where they help lower pH. This acidification can be monitored using pH-sensitive indicators, optical sensors, and electrochemical biosensors, providing a direct measure of microbial metabolic activity and spoilage progression [70].

VOCs such as aldehydes, ketones, and alcohols are also produced during lipid oxidation and microbial metabolism. Electronic nose systems and advanced spectroscopic techniques commonly detect these compounds by analyzing complex volatile patterns rather than individual compounds. VOC profiling is widely used to rapidly classify fresh versus spoiled food products [69].

Detecting these spoilage-related metabolites, especially volatile gases and VOCs, has driven the development of a wide range of sensing technologies. Gas sensors that detect compounds such as NH_3_, H_2_S, and VOCs are increasingly used for real-time food-quality monitoring [211]. Monitoring these gas concentrations enables not only freshness assessment but also identification of spoilage stages and sources, allowing timely intervention to prevent further deterioration.

To support these applications, researchers have explored various sensing materials, including nanostructured metal oxides and conducting polymers [56,212]. Among these, semiconducting metal oxides such as WO_3_, ZnO, SnO_2_, and TiO_2_ are particularly prominent due to their favorable electrical properties, high surface area, and cost-effectiveness [213]. Advances in nanotechnology have further enhanced sensor performance by increasing the active surface area available for gas interaction, thereby improving sensitivity and response time.

Recent research has focused on optimizing key sensor performance parameters, including sensitivity, selectivity, stability, and response time. In parallel, advances in data analysis and signal processing have enabled the integration of chemiresistive gas sensors into intelligent systems, facilitating real-time monitoring and more accurate decision-making in food quality management [214].

Overall, detecting microbial metabolites relies on a combination of chemical, electrochemical, optical, and biosensing technologies. These systems convert biochemical changes caused by microbial activity into measurable signals, enabling real-time, non-destructive assessment of food spoilage and quality deterioration.

### 4.4. Multi-Analyte and Sensor Arrays (Electronic Nose/Tongue Systems)

Although single-analyte sensors can provide valuable information regarding specific spoilage markers, food degradation typically involves the simultaneous production of multiple chemical compounds. Consequently, multi-analyte sensing approaches based on sensor arrays have emerged as a more comprehensive strategy for monitoring complex volatilomic signatures in food systems [122]. By detecting patterns of chemical responses rather than individual compounds, these systems can characterize complex aroma profiles associated with food freshness, fermentation, and spoilage progression.

Within this context, e-nose systems are among the most widely investigated technologies for multi-analyte detection. These devices mimic the biological olfactory system by combining arrays of partially selective gas sensors with pattern-recognition algorithms that can identify complex odor profiles [215]. Each sensor within the array exhibits a different response pattern toward specific VOCs, and the collective signal pattern, often referred to as an “odor fingerprint”, can be analyzed using multivariate statistical techniques or machine learning algorithms to classify food quality or spoilage levels.

From a technological perspective, a typical e-nose architecture comprises three main components: a gas sensor array, a data acquisition system, and a signal processing module that performs pattern recognition and classification [216]. Common sensing elements include MOS sensors, conducting polymer sensors, quartz crystal microbalance (QCM) sensors, and surface acoustic wave (SAW) sensors, each providing complementary sensitivity to different classes of volatile compounds [112]. The diversity of sensing materials enhances the system’s discriminatory power, enabling differentiation among freshness stages, microbial contamination levels, and product authenticity.

Recent research has demonstrated the effectiveness of electronic noses for monitoring spoilage in various food matrices, including meat, fish, dairy products, fruits, and fermented beverages. For instance, sensor arrays have been used to detect early-stage meat spoilage by monitoring microbial volatile metabolites generated during storage, often detecting spoilage earlier than conventional microbiological methods [217]. Similarly, e-nose systems have been applied to monitor fermentation dynamics and flavor development in bread and alcoholic beverages by tracking changes in volatile compound profiles during processing [218].

In parallel, research has increasingly focused on improving the analytical performance of sensor arrays through hybrid sensing architectures. Studies conducted by Meléndez and coworkers have contributed significantly to the development of multisensor platforms for food analysis. Integrated sensor arrays combining metal-oxide and electrochemical sensors have been used to detect volatile compounds emitted by agricultural products, demonstrating the potential of hybrid sensing architectures to improve sensitivity and selectivity in complex headspace environments [196]. Such systems are particularly valuable for real-time monitoring of ripening, spoilage, and storage conditions.

While electronic noses (e-noses) primarily detect volatile compounds, complementary technologies monitor non-volatile chemical species in liquid matrices. Electronic tongue (e-tongue) systems use arrays of electrochemical sensors to detect dissolved compounds, including organic acids, phenolic compounds, and fermentation metabolites. These devices are widely used to assess beverages such as wine, beer, and fermented dairy products, where subtle variations in chemical composition influence sensory quality and product authenticity [124]. When integrated with e-nose platforms, these systems enable comprehensive evaluation of volatile and non-volatile chemical signatures, providing a multidimensional characterization of food quality.

The effectiveness of these multisensor systems relies heavily on advanced data analysis techniques that extract meaningful information from complex sensor signals. Multivariate statistical tools such as principal component analysis (PCA), linear discriminant analysis (LDA), and partial least squares (PLS) are commonly used to interpret sensor responses and visualize differences between sample classes. More recently, machine learning techniques, including support vector machines (SVMs), artificial neural networks (ANNs), and deep learning models, have demonstrated improved performance on complex classification tasks involving large sensor datasets [122].

Several studies have highlighted the importance of integrating sensor arrays with chemometric and machine-learning approaches to interpret volatilomic patterns associated with food ripening and spoilage. For example, Modesti and co-workers have demonstrated that portable sensor arrays combined with multivariate analysis can successfully identify characteristic odor signatures and improve the predictive capability of sensor-based quality monitoring systems [121].

Despite their significant potential, sensor array systems still face several challenges that limit large-scale industrial adoption. These include sensor drift, cross-sensitivity to environmental parameters such as humidity and temperature, and limited standardization of sampling protocols and data-processing methodologies [122]. Nevertheless, ongoing progress in sensor materials, calibration strategies, and artificial intelligence is expected to enhance the robustness and reliability of electronic nose and tongue technologies, thereby supporting their integration into intelligent food-monitoring systems and smart packaging solutions.

**Figure 1:**
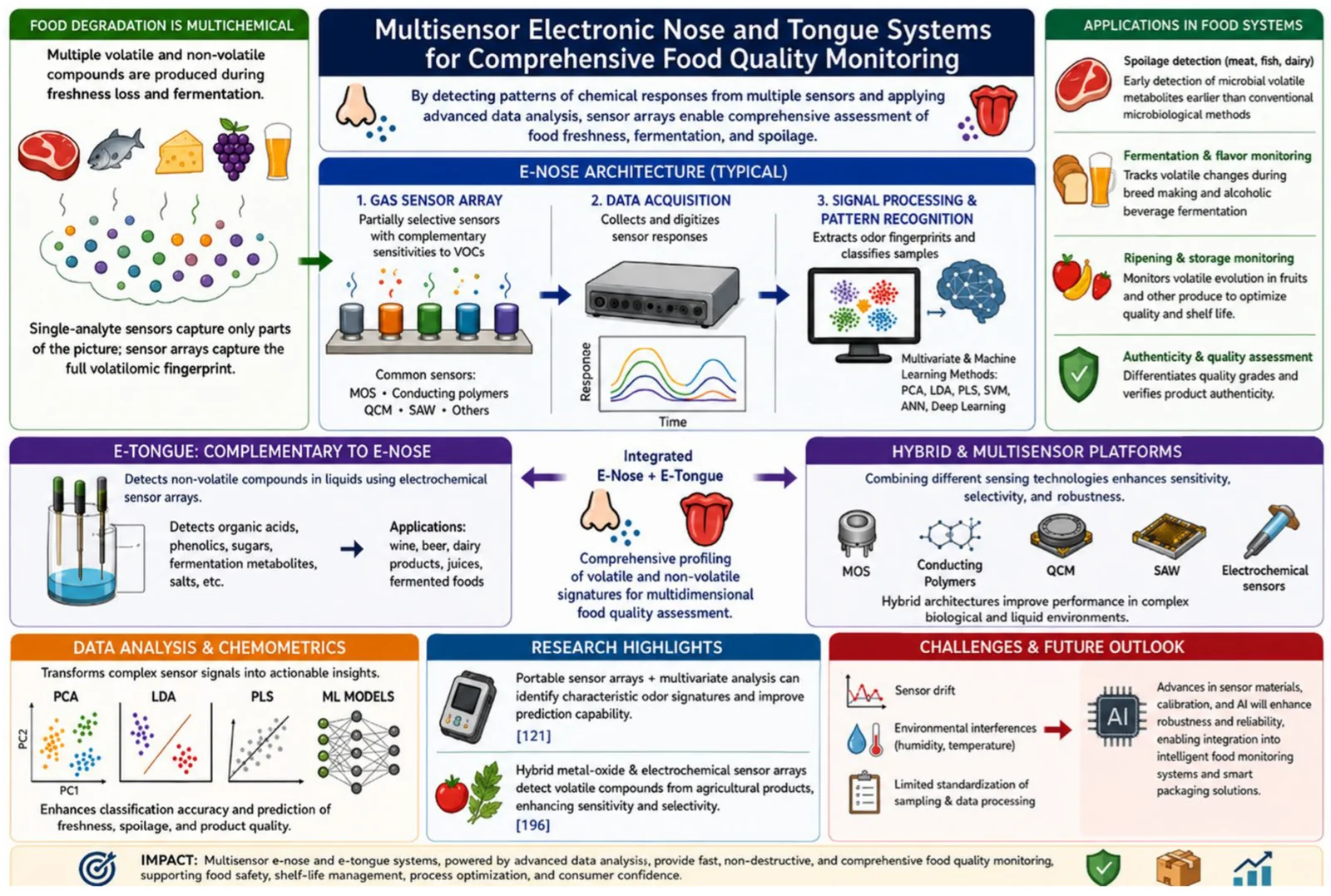
Schematic representation of multisensor e-nose and e-tongue systems for comprehensive food quality monitoring [121,196].

Figure 1 summarizes a multi-analyte sensor-array system. In the figure, Sensor arrays composed of partially selective elements (e.g., metal-oxide semiconductors, conducting polymers, QCM, and SAW sensors) detect complex patterns of volatile organic compounds (VOCs), generating characteristic odor fingerprints. These signals are acquired and processed using multivariate statistical methods and machine learning algorithms (e.g., PCA, LDA, PLS, SVM, ANN) to classify food freshness, fermentation dynamics, and spoilage progression. Complementary e-tongue systems detect non-volatile compounds in liquid matrices. Hybrid sensor architectures integrate these approaches to enhance sensitivity and selectivity, enabling applications across diverse food systems. Despite challenges such as sensor drift and environmental interference, advances in materials and artificial intelligence support the development of robust, real-time food-monitoring technology.

## 5. Applications in Fermented Food Products

Fermented foods are widely appreciated in contemporary food systems for their extended shelf life, sensory complexity, and, in many cases, beneficial health properties [219,220]. At the same time, they are analytically complex matrices because dynamic microbial activity shapes their quality and generates a wide range of metabolites, including microbial volatile organic compounds (MVOCs), organic acids, sulfur compounds, and biogenic amines. Many of these compounds are associated with both desirable fermentation processes and undesirable deviations, making it difficult to clearly distinguish spoilage. Consequently, real-time monitoring is particularly challenging, and there is growing interest in smart chemical sensors that can continuously and non-destructively track biochemical changes, rather than relying solely on delayed laboratory testing at fixed checkpoints [221,222].

### 5.1. Dairy (Yogurt, Cheese)

The Mediterranean diet recommends the moderate daily consumption of fermented dairy products, highlighting their recognized nutritional value [223]. These products are rich in essential nutrients, including calcium, proteins, vitamins, and minerals, as well as microorganisms with probiotic potential that contribute to human health [224,225]. As a result, fermented dairy products are a fundamental component of traditional diets across diverse regions worldwide. They are produced from the milk of various animal species through spontaneous fermentation or back-slopping, processes that underpin their biochemical complexity and distinctive sensory characteristics [226].

Fermented dairy products, including yogurt and cheese, are complex, dynamic biochemical systems in which microbial metabolism plays a dual role, contributing to both desirable organoleptic properties and potential spoilage pathways [227,228]. Therefore, these matrices are among the most demanding for sensor-based monitoring. In yogurt, acidification, starter activity, and aroma formation evolve rapidly over a relatively short production window [229]. Cheese is even more complex, since ripening introduces proteolysis, lipolysis, moisture gradients, surface microbiota, and, depending on the product, long maturation periods [230]. As a result, a sensor signal in dairy rarely reflects a single biochemical pathway; more often, it captures a superposition of fermentation progress, aroma development, and potential quality loss. This complexity makes dairy products particularly relevant to developing smart sensing approaches, where rapid, non-destructive monitoring is advantageous, yet interpretation remains highly context-dependent [221,231].

In yogurt, recent research suggests that the field is moving beyond simple endpoint pH control toward multi-parameter monitoring and predictive modeling frameworks. Hristovski et al. [232] showed that microbial potentiometric sensors could monitor kefir-facilitated milk fermentation in real time, with signal characteristics correlating strongly with inoculum mass and fermentation completion time. Although this study focused on kefir rather than conventional yogurt, it remains relevant because it shows that electrochemical signatures from such sensors can continuously track biologically mediated fermentation dynamics rather than only retrospectively. Complementary approaches based on volatile analysis have also been explored. E-nose systems combined with machine-learning algorithms have demonstrated strong performance in classifying yogurt aroma profiles, enabling discrimination based on quality attributes and storage conditions [233]. This is a useful step for quality discrimination, though not a direct assay for spoilage. Moreover, e-nose and e-tongue systems help maintain conditions conducive to microbial growth and activity, which is essential for ensuring consistent product quality in products such as yogurt [234] and cheese derivatives [199]. Beyond gas-phase sensing, researchers have also investigated electronic tongue systems for yogurt classification, providing complementary information on dissolved compounds and taste-related attributes. When coupled with machine learning, these systems have demonstrated the ability to discriminate yogurt samples based on compositional and quality differences. However, challenges related to calibration, repeatability, and matrix effects remain [123].

More recent studies show an evolution from classification models to predictive models. Saetae [235] proposed a machine-learning framework to predict microbial growth and acidification during yogurt fermentation, demonstrating marked reductions in prediction error compared with classical models. Ray et al. [236] moved further by integrating pH, electrical conductivity, temperature, and total dissolved solids into an AI-driven real-time monitoring and predictive control system for yogurt fermentation. This is an important concept. In fermented dairy, the first practical role of smart sensors may not be direct spoilage labeling, but early detection of abnormal fermentation trajectories likely to lead to defective quality or instability. However, this remains challenging because milk composition, starter combination, formulation, and processing temperature strongly affect sensor response patterns, limiting model transferability between plants or even between formulations of the same product [235,236].

Recent work on co-fermented yogurt systems provides additional evidence. Huang et al. [237], using an electronic nose, electronic tongue, and gas chromatography-ion mobility spectrometry (GC-IMS), showed that co-fermentation altered the aromatic profile of yogurt, namely aldehydes, ketones, and alcohols. This type of study matters because it highlights a key issue in fermented-food analysis: changes in volatile compound profiles that are not necessarily indicative of spoilage.

For cheese, studies are more clearly tied to spoilage-associated chemistry, particularly biogenic amines. Histamine and tyramine are especially relevant because they can accumulate during ripening or under suboptimal hygienic and storage conditions [238,239]. Recent investigations, acknowledging the limitations of conventional techniques, such as limited specificity, costly instrumentation, time-intensive procedures, low sensitivity, and the requirement for trained personnel, have increasingly explored alternative analytical strategies. In this context, biosensors have emerged as promising tools, offering a combination of sensitivity, robustness, and technological adaptability. These systems provide faster, more economical, and user-friendly approaches to detecting biogenic amines, making them attractive candidates for practical applications [240]. A recent example is a dual-template molecularly imprinted fluorescence sensor for the simultaneous detection of histamine and tyramine in cheese. The sensor achieved a limit of detection of 14.57 μmol/L, recovery rates of 99.46–103.02%, and relative standard deviations below 6.16% in cheese samples, indicating that selective, relatively rapid detection in a real fermented dairy matrix is feasible. The main strength of this type of system lies not only in its sensitivity but also in its attempt to address matrix complexity through tailored recognition chemistry [241]. In parallel, Givanoudi et al. [242] showed that biosensors and chemosensors for biogenic amines are progressing rapidly, but specificity, matrix interference, stability, and practical deployment remain common limitations.

Meenakshi et al. [231] reviewed emerging non-destructive testing techniques (NDTTs) for detecting microbial contamination in cheese, including hyperspectral imaging, spectroscopic approaches (near-infrared, FTIR, fluorescence, and surface-enhanced Raman), and e-noses. These methods rely on the product’s optical and chemical properties [243]. When combined with advanced chemometric tools, NDTTs show strong potential for real-time monitoring of microbial contamination in the cheese industry. However, the inherent heterogeneity of cheese, both in its structure and microbiota, means that changes linked to maturation can partly overlap with those associated with spoilage, posing a significant challenge to the practical application of these techniques [231].

### 5.2. Meat Fermentation (Sausages)

Fermented meat products, particularly sausages, result from a complex interaction between microbial activity, biochemical transformations, and processing conditions, in which fermentation, drying, and curing collectively shape the final quality attributes [244,245]. From a biochemical perspective, fermented meat systems feature highly dynamic metabolic processes, including proteolysis, lipolysis, carbohydrate fermentation, and amino acid catabolism [246], which influence flavor, palatability, and the formation of volatile compounds that contribute to aroma [247,248,249]. Additives and environmental conditions also drive flavor-component formation [250]. However, the same metabolic processes can also lead to the excessive accumulation of biogenic amines, undesirable volatile compounds, and oxidative byproducts when the system deviates from the intended fermentation pathway [249,251,252].

Recent studies show that smart chemical sensors, particularly e-nose systems, have strong potential to monitor changes in fermented-sausage quality by detecting volatile organic compounds. Shao et al. [252] examined changes in free amino acids, biogenic amines, and volatile compounds in fermented sausages inoculated with *Lactiplantibacillus plantarum* and *Staphylococcus simulans*. Using ultra-performance liquid chromatography, gas chromatography-ion mobility spectrometry (GC-IMS), and measurements with e-nose and e-tongue, they showed that the inoculated groups exhibited lower pH and carbonyl levels, and that *L. plantarum*, in particular, reduced free amino acids and biogenic amines, while promoting alcohols, ketones, and alkenes associated with a more desirable aroma development.

Chen et al. [246] used a combined e-nose and e-tongue platform to characterize the flavor profiles of fermented sausages in response to changes in formulation and composition, highlighting the system’s sensitivity to variations in both volatile and non-volatile compounds.

Integrating high-throughput sequencing with gas chromatography-ion mobility spectrometry showed that volatile profiles could be correlated with microbial community structure and product characteristics. This allowed discrimination between different sausage types and processing conditions [253].

From an application standpoint, sensors focused on biogenic amines show great promise. Givanoudi et al. [242] discuss a variety of sensor technologies, including receptor-free chemosensors based on electrochemical detection and biosensors incorporating selective recognition elements, such as enzymes, antibodies, aptamers, and molecularly imprinted polymers (MIPs). The authors emphasize that these sensors offer significant advantages over conventional chromatographic methods, enabling rapid in situ analysis across the entire food chain. Zamfir et al. [254] further advance this line of work by demonstrating that portable optoelectrochemical biosensors can detect histamine and putrescine produced by food-associated microorganisms. Although the work was not limited to fermented sausage matrices, it is relevant as a translational case study because it advances the development of portable, microbiologically grounded detection of spoilage-linked amines.

### 5.3. Beverages (Wine, Beer)

Fermented beverages present a different challenge because the matrix is fluid, the volatile phase is highly informative, and many relevant defects emerge at low sensory thresholds. In wine, spoilage generally involves the accumulation of acetic acid, volatile phenols associated with Brettanomyces, sulfur-related defects, or compounds associated with cork taint [255,256]. In beer, relevant deviations include lactic acid imbalance, microbial contamination, and aroma changes associated with oxidation or the formation of undesirable flavors [257].

Compared with dairy products or sausages, beverage systems are particularly well suited to headspace-based detection, which partly explains why electronic noses have been studied so extensively in enology and brewing. Indeed, smart chemical sensors, particularly e-noses, have emerged as promising tools for rapid, non-destructive assessment of beverage quality [258]. These systems rely on partially selective sensor arrays, such as metal oxide semiconductors, conductive polymers, or quartz microbalances, which generate characteristic response patterns when exposed to complex mixtures of volatile organic compounds [259]. Recent developments in nanostructured sensor arrays, including SWCNT-based electronic noses functionalized with metal phthalocyanines, have further shown that e-nose systems can discriminate complex wine matrices and support beverage authentication and quality assessment [260].

As several recent reviews discuss, electronic nose technologies can effectively mimic human olfactory perception. They provide reproducible, quantitative results, making them an attractive alternative to conventional sensory evaluation and chromatographic techniques [122,261,262,263]. Furthermore, these technologies can be applied at all stages of production, not just in the final wine evaluation [262]. Recent studies have shown that electronic noses based on metal oxide semiconductors (MOS) and quartz crystal microbalance (QCM) can detect volatile compounds associated with spoilage, including defects linked to acetic acid and trichloroanisole [264,265], as well as phenolic deviations associated with *Brettanomyces* spp. [266]. Table 5 presents a range of e-nose applications, sensor types, and chemometric approaches for evaluating wine parameters.

The e-tongue has progressively been explored as a complementary tool for detecting wine defects, particularly those affecting the non-volatile components and gustatory balance of wine [280,281]. Unlike electronic nose systems, the e-tongue uses arrays of electrochemical sensors (potentiometric, voltammetric, or impedimetric) in conjunction with multivariate analysis [124,282]. Its high cross-sensitivity and stability make it particularly well suited for pattern-recognition applications, enabling a comprehensive evaluation of the wine matrix. Several studies demonstrate its ability to detect defects in red and white wines at an early stage [280,281,283]. These authors’ research goes further, suggesting that the electronic tongue could be an effective tool for the early monitoring of oxidative and microbiological changes, complementing or even anticipating traditional sensory analysis. Furthermore, the e-tongue could differentiate between grape varieties, geographical origins, harvest years, and aging techniques [124].

Recently, combining e-tongue with machine learning algorithms (e.g., support vector machines or artificial neural networks) has significantly improved the accuracy of defective wine classification, even in complex matrices [258].

Researchers have integrated electronic noses and tongues to evaluate technological factors associated with wine, such as the influence of corks on oxygen levels and wine antioxidant capacity [284]. Meanwhile, combining these systems with an ‘electronic eye’ has enabled the development of a multisensory electronic panel that can distinguish wines produced using the same technology but from different varieties, based on their organoleptic characteristics [285].

In the brewing industry, smart chemical sensors are used, following principles similar to those applied in winemaking. Beer spoilage is commonly associated with microbial contamination [e.g., lactic acid bacteria], oxidation, or process-related deviations. These can form undesirable flavor compounds, such as diacetyl, dimethyl sulfide, and aldehydes (e.g., trans-2-nonenal), which contribute to off-flavors [286].

Researchers developed an enzymatic lactate sensor and applied it to craft beers to quantify lactic acid. It is positioned as a fast alternative to laborious laboratory methods and is relevant for the early detection of acidification/contamination [287]. Electrochemical biosensors have also shown promising results in quality monitoring and deterioration detection. Almeida et al. [288] developed a simple, versatile, low-cost glass carbon electrode (GCE) modified with multi-walled carbon nanotubes (MWCNTs) for sensitive, selective detection of tyrosinase (TYR) at pH 9.2. Compared with high-performance liquid chromatography (HPLC) results, the sensor’s potential to quantify tyramine in aqueous food matrices without extensive sample preparation was highlighted. According to Majer-Baranyi et al. [289], enzymatic and microbial sensors have been successfully used to quantify compounds directly associated with spoilage processes, such as ethanol, fermentable sugars, and organic acids. Enzyme-based sensors are widely used to control fermentative parameters and detect metabolic deviations during storage [112]. Amperometric sensors have also been used to monitor compounds such as lactic acid and acetic acid, which are often associated with contaminating microbial activity. These systems show good sensitivity and short response times, with potential applications in online control that contribute to faster, more objective assessment of beer’s microbiological stability [289].

The use of e-nose systems for detecting beer defects has become increasingly relevant as a quick, non-destructive quality-control method, particularly because they can analyze volatile compounds associated with off-flavors [290].

Studies demonstrate that when combined with machine learning algorithms, e-nose systems can effectively discriminate among beers with experimentally induced defects, including different types and levels of off-flavors, achieving classification rates of 95–98%. This approach enables detection and quantification of defect severity, which is particularly relevant for industrial applications [291]. In addition to directly detecting defects, these devices can monitor product stability. They can distinguish between fresh and aged beers, predict shelf life based on the evolution of the volatile profile, and characterize beers from different breweries and brands [292,293].

Overall, the e-nose appears to be a promising tool for the early detection of brewing defects, offering speed, sensitivity, and the potential for integration into online control systems. However, its effectiveness depends heavily on the calibration and robustness of the chemometric models used.

## 6. Applications in Non-Fermented Food Products

### 6.1. Fresh Meat and Seafood

Fresh meat and seafood are among the most challenging non-fermented food matrices for shelf-life monitoring because of their rapid perishability, high water activity, and rich protein and lipid content [70,208,294]. Spoilage processes are driven by a combination of endogenous enzymatic activity and the proliferation of specific spoilage organisms (SSOs), producing a complex mixture of volatile and non-volatile metabolites that can serve as chemical markers for sensor-based detection [7,51,295].

In fresh meat, post-mortem biochemical processes, including proteolysis and lipolysis, generate precursors that microorganisms further metabolize into spoilage-related compounds [70,296,297]. Aerobic storage conditions typically favor *Pseudomonas* spp., whereas vacuum or modified-atmosphere packaging (MAP) promotes lactic acid bacteria and *Brochothrix thermosphacta* [298]. These microbial communities produce characteristic metabolites such as ammonia, hydrogen sulfide, short-chain fatty acids, aldehydes, ketones, and biogenic amines including putrescine and cadaverine [299,300]. The accumulation of these compounds correlates with total viable counts (TVC) and sensory rejection thresholds [70].

In seafood, spoilage is even more rapid due to the presence of trimethylamine oxide (TMAO), which is reduced by spoilage bacteria (e.g., *Shewanella putrefaciens*, *Photobacterium phosphoreum*) into trimethylamine (TMA), a key indicator of fish deterioration [301]. Additional markers include dimethylamine (DMA), ammonia, sulfur-containing compounds, and a wide range of microbial volatile organic compounds (MVOCs) [302]. These compounds are commonly associated with total volatile basic nitrogen (TVB-N), a standard parameter used in seafood quality assessment [303].

A wide range of smart chemical sensors has been developed to target these spoilage markers, leveraging different transduction mechanisms: Chemiresistive sensors based on metal oxide semiconductors (MOS) (e.g., SnO_2_, ZnO, TiO_2_) and conducting polymers (e.g., polyaniline, polypyrrole) are among the most widely studied platforms [111,304]. These sensors detect changes in electrical resistance when they interact with volatile compounds such as ammonia and amines. Researchers have incorporated nanostructured materials, including graphene and carbon nanotubes (CNTs), to increase surface area, sensitivity, and response time, achieving detection limits in the ppb–ppm range [305].

Optical and colorimetric sensors have gained particular attention for their suitability in intelligent packaging [116,306]. These systems typically rely on pH-sensitive dyes (e.g., bromocresol green, methyl red) or natural pigments such as anthocyanins, which change color visibly in response to alkaline spoilage metabolites [307]. More advanced approaches include porphyrins, metalloporphyrins, and metal–organic frameworks (MOFs), which can selectively interact with specific VOCs, enabling multiplexed detection [308].

Electrochemical sensors and biosensors provide high selectivity through biological recognition elements such as enzymes (e.g., amine oxidases), antibodies, or aptamers [180]. These systems can quantify specific analytes, such as histamine or TMA, with high accuracy and have been applied particularly to seafood quality monitoring, where histamine accumulation is associated with food safety risks (e.g., scombroid poisoning) [309].

E-noses, consisting of arrays of partially selective sensors, are increasingly used to capture the overall volatile fingerprint of meat and seafood [69,310]. When combined with chemometric tools and machine learning algorithms (e.g., principal component analysis, support vector machines, artificial neural networks), these systems enable classification of freshness levels, prediction of shelf life, and discrimination between storage conditions or packaging types. Recent studies have further demonstrated their practical applicability under real distribution-chain conditions, where portable e-nose systems have been successfully used to establish freshness warnings and control limits for seafood products, showing good agreement with conventional microbiological analyses [311]. One of the most promising applications of smart chemical sensors in fresh meat and seafood is their integration into intelligent packaging [167,312]. Colorimetric indicators embedded in packaging films or labels provide real-time, non-destructive visual feedback on product freshness that consumers can interpret directly without instrumentation [313]. These systems are particularly attractive due to their low cost, ease of use, and compatibility with existing packaging technologies.

More advanced implementations include wireless sensor systems and RFID-based platforms, which enable remote monitoring of food quality throughout the supply chain [314]. These systems can integrate environmental sensors (e.g., temperature, humidity) with chemical sensing elements, allowing for more accurate prediction of spoilage under dynamic storage conditions.

Despite significant technological progress, several challenges remain for the widespread adoption of smart sensors in fresh meat and seafood applications. High humidity, temperature fluctuations, and complex food matrices can interfere with sensor performance, leading to signal drift, cross-sensitivity, and reduced reproducibility [315]. In addition, the variability in microbial communities and spoilage pathways across different products and storage conditions complicates the identification of universal markers [54].

From a practical standpoint, developing food-grade, non-toxic, and biodegradable sensing materials is essential for integration into packaging systems [315]. Regulatory compliance, cost-effectiveness, and scalability are also critical factors that must be addressed to facilitate commercialization.

Future research is increasingly focused on developing hybrid systems that combine multiple sensing modalities with advanced data analytics. Integrating sensor arrays with artificial intelligence and Internet of Things (IoT) technologies is expected to significantly enhance predictive capabilities, enabling real-time decision-making and optimization of cold chain logistics [316].

### 6.2. Fruits and Vegetables

Fresh fruits and vegetables differ fundamentally from meat and seafood in that they remain physiologically active after harvest, with postharvest quality largely governed by ongoing metabolic processes such as respiration, transpiration, ripening, senescence, and enzymatic activity. [317,318]. These processes, along with high water activity and tissue fragility, make fresh produce particularly susceptible to rapid quality deterioration, even without significant microbial growth [319].

Spoilage in fruits and vegetables therefore results from a tight interplay between physiological senescence and microbial activity, with each component’s relative contribution depending on commodity type, maturity stage, and storage conditions. Senescence-related processes include chlorophyll degradation, softening due to cell wall polysaccharide breakdown, membrane lipid peroxidation, and shifts in primary metabolism, leading to losses in firmness, color, and nutritional value [320]. In parallel, opportunistic microorganisms such as *Pseudomonas*, *Erwinia*, and fungal pathogens including *Botrytis cinerea* and *Penicillium* spp. accelerate tissue degradation through enzymatic maceration and secondary metabolite production [319,321].

A key distinguishing feature of fruit and vegetable spoilage is the central role of plant-derived volatile organic compounds (VOCs), many of which are intrinsic to normal ripening but become dysregulated during over-ripening and decay. In climacteric fruits, the transition from ripening to senescence is associated with increased respiration and ethylene production, accompanied by shifts in aroma profiles, including changes in esters, alcohols, aldehydes, and terpenes [322]. Ethylene acts as a master regulator of ripening, and its accumulation not only reflects physiological status but can also accelerate deterioration in surrounding produce, making it a critical indicator for monitoring and management [323].

Under stress or suboptimal storage conditions (e.g., low-oxygen or high-CO_2_ atmospheres), fruits may shift towards anaerobic metabolism, leading to the accumulation of ethanol and acetaldehyde, which are commonly associated with off-flavors and reduced quality [317,324]. In addition, lipid oxidation and membrane degradation processes contribute to the formation of compounds such as hexanal and other C6 volatiles, which are widely recognized as markers of tissue disruption [325].

In vegetables, particularly leafy greens and minimally processed products, spoilage is often characterized by rapid senescence combined with microbial proliferation. The emission of sulfur-containing compounds, aldehydes, ketones, and organic acids reflects both enzymatic degradation pathways and microbial metabolism [319]. Unlike fruits, where sweetness and aroma are key quality attributes, visual appearance (e.g., color retention) and texture dominate, closely linked to chlorophyll degradation and water loss [326].

Another important aspect specific to fruits and vegetables is the strong influence of external environmental factors on deterioration dynamics. Temperature, relative humidity, and atmospheric composition (O_2_, CO_2_, ethylene) not only modulate metabolic rates but also affect VOC emission patterns, complicating the interpretation of spoilage markers [327]. Moreover, high biological variability across species, cultivars, and ripening stages adds complexity, limiting the applicability of universal indicators.

From a sensing perspective, these characteristics imply that fresh-produce monitoring strategies must account for both physiological and microbiological signals, often at lower analyte concentrations and with greater temporal variability than in animal-based products [324]. Consequently, multi-parameter approaches that simultaneously track key indicators such as ethylene, ethanol, and stress-related volatiles are increasingly emphasized, rather than relying on single-compound detection [323,328].

Despite recent advances, significant challenges remain, particularly in distinguishing between normal ripening and early spoilage, as both processes share overlapping metabolic signatures. Improving selectivity and context-aware interpretation of sensor outputs—potentially through integration with environmental data and predictive models—will be essential to enhancing reliability. Future developments are expected to combine high-sensitivity detection with advanced data analytics to better capture the dynamic nature of postharvest physiology and reduce losses along fresh produce supply chains [329,330].

### 6.3. Packaged and Processed Foods

Packaged and processed foods encompass a highly diverse group of products, including ready-to-eat meals, dairy products, bakery goods, and minimally processed foods, whose shelf life and quality largely depend on the combined effects of processing technologies, formulation, and packaging conditions [331]. Unlike fresh products, spoilage dynamics in these matrices are strongly influenced by prior treatments such as thermal processing, fermentation, drying, or the addition of preservatives, which alter both the native microbiota and the biochemical pathways that lead to deterioration [328].

A defining feature of these systems is the prevalence of sub-lethally injured or stress-adapted microorganisms that survive processing and subsequently recover during storage [252]. These include spore-forming bacteria (e.g., *Bacillus* spp., *Clostridium* spp.) and psychrotrophic organisms that can grow under refrigerated conditions, particularly in ready-to-eat and dairy products [299,332]. In addition, post-processing contamination during handling and packaging represents a critical route for spoilage and food safety risks, especially in minimally processed and ready-to-eat foods [6].

The metabolic activity of these microbial communities produces a distinct set of spoilage-related compounds, often differing from those observed in fresh products. In dairy systems, for example, proteolysis and lipolysis produce free fatty acids, ketones, aldehydes, and sulfur compounds that contribute to off-flavors and rancidity [199]. In bakery and cereal-based products, spoilage is frequently associated with fungal growth (e.g., *Fusarium*, *Penicillium*, *Aspergillus*, and *Alternaria*) and the production of alcohols, organic acids, and mycotoxins, particularly under high-humidity conditions [333,334]. Ready-to-eat meals and processed foods may also accumulate organic acids, biogenic amines, and volatile sulfur compounds, depending on formulation and storage conditions [70].

Another important aspect specific to packaged foods is the interaction between the product and the packaging environment. Modified atmosphere packaging (MAP) and vacuum packaging are widely used to extend shelf life by altering oxygen and carbon dioxide levels; however, these conditions also select for specific microbial communities and metabolic pathways, leading to characteristic volatile profiles [335]. In parallel, physicochemical changes such as lipid oxidation, Maillard reactions, and moisture migration contribute to quality degradation independently of microbial activity, generating compounds such as aldehydes, furans, and other reactive carbonyl species [336].

In this context, spoilage monitoring in packaged and processed foods relies on detecting a broader, often more complex set of indicators, encompassing both microbial metabolites and chemical degradation products [149]. Key target analytes include carbon dioxide (CO_2_) as an indicator of microbial respiration; oxygen (O_2_) levels that reflect package integrity; ethanol and organic acids associated with fermentation; and specific volatiles such as hexanal and 2,3-butanedione, which are linked to lipid oxidation and flavor deterioration [149,337].

A further distinguishing feature is the importance of package integrity and headspace composition as indirect indicators of product quality. Changes in internal gas composition, due to microbial activity or packaging failure, can precede sensory spoilage and therefore provide an early warning signal. In this sense, monitoring strategies for packaged foods often integrate chemical sensing with physical parameters such as pressure, gas permeability, and seal integrity [338].

However, the heterogeneity of processed food systems poses significant challenges for identifying universal spoilage markers. Variations in formulation (e.g., fat, salt, sugar content), processing intensity, and storage conditions result in highly product-specific deterioration pathways [339]. Moreover, additives and preservatives can mask or delay the formation of typical spoilage indicators, complicating interpretation [30,340].

Future developments in this area are expected to focus on tailored sensing approaches adapted to specific product categories, combined with predictive models that incorporate formulation, processing history, and storage conditions. Integrating multi-parameter monitoring systems that simultaneously assess gas composition, volatile profiles, and environmental variables will be essential to improve reliability and support real-time quality management in increasingly complex food supply chains [30,116].

### 6.4. Smart Packaging Applications

Smart packaging represents a paradigm shift from passive containment systems to interactive and responsive platforms that can monitor, communicate, and, in some cases, modulate food quality throughout the supply chain [167,341]. These systems are generally classified into two complementary categories: intelligent packaging, which provides information about the product’s condition, and active packaging, which interacts with the internal environment to extend shelf life [167,342].

A central application of smart packaging lies in its ability to translate complex physicochemical and biological changes into simple, actionable outputs [343]. This is particularly relevant for perishable foods, where real-time freshness information can significantly reduce uncertainty associated with fixed expiry dates. Time–temperature indicators (TTIs), for example, integrate thermal history into a cumulative signal, enabling more accurate assessment of product quality under fluctuating storage conditions [344]. Similarly, gas indicators that target O_2_ or CO_2_ provide indirect information on microbial activity and package integrity, enabling early detection of deviations from optimal storage conditions [345].

More advanced implementations involve multifunctional labels that can simultaneously monitor several parameters, such as temperature abuse, gas composition, and the presence of specific metabolites [346]. These integrated systems move beyond single-analyte detection and enable a more holistic evaluation of product status, which is particularly valuable in complex supply chains where multiple stressors act simultaneously [347]. In this context, combining sensing elements with data processing units enables the generation of predictive indicators rather than simple threshold-based responses [348].

Another important development is incorporating smart packaging into digital infrastructures. Integrating radio-frequency identification (RFID), near-field communication (NFC), and wireless sensor networks enables continuous data acquisition and remote access to quality-related information [349,350]. These technologies facilitate traceability and transparency across the food chain, allowing stakeholders to monitor product condition in real time and respond proactively to deviations. Integrating packaging systems with cloud-based platforms and data analytics tools further enhances their functionality, enabling predictive shelf-life modeling and optimizing logistics [351].

From a consumer perspective, smart packaging offers a more intuitive way to assess food quality. Visual indicators, digital readouts, or smartphone-enabled interactions can improve trust and reduce food waste by providing clearer information about the product’s actual condition rather than conservative “best-before” dates [352]. This shift towards condition-based shelf-life assessment aligns with broader sustainability goals and regulatory efforts to minimize food losses.

Despite these advantages, several constraints limit large-scale implementation. Integrating sensing and communication components into packaging materials must balance performance with cost, scalability, and compatibility with existing manufacturing processes [353]. In addition, data standardization, interoperability, and cybersecurity become increasingly relevant as packaging systems become digitally connected [354]. Regulatory frameworks also need to evolve to address novel materials and embedded electronics in food-contact applications.

Future smart packaging systems are expected to evolve into fully integrated platforms that combine sensing, data processing, and decision-support capabilities. The convergence of advanced materials, flexible electronics, and artificial intelligence will enable adaptive systems capable not only of monitoring but also of responding dynamically to changes in product condition. Such developments could transform food quality management from a reactive to a predictive, data-driven process, ultimately enhancing food safety, reducing waste, and improving supply chain efficiency [345,348,351,352].

Smart chemical sensors in both fermented and non-fermented foods show broad potential for real-time quality monitoring. However, important differences exist between these food categories. In non-fermented foods, sensing strategies primarily focus on the early detection of spoilage-related biomarkers, including ammonia, hydrogen sulfide, trimethylamine, biogenic amines, and spoilage-associated volatile organic compounds. In contrast, fermented foods pose a more complex analytical challenge, as sensors must distinguish metabolites generated during desirable fermentation processes from those associated with quality deterioration or microbial contamination. Consequently, while similar sensing platforms may apply to both food categories, fermented food systems often require more sophisticated data interpretation, greater selectivity, and the integration of chemometric or artificial intelligence tools to distinguish normal fermentation dynamics from spoilage events. This comparison highlights the importance of tailoring sensor design and data-analysis strategies to each food matrix’s specific biochemical characteristics.

## 7. Data Processing and Artificial Intelligence

Smart chemical sensors often generate complex datasets containing responses to multiple spoilage-related compounds. Consequently, data analysis tools have become essential for converting sensor outputs into meaningful information regarding food freshness, spoilage status, and shelf-life estimation. In food spoilage applications, chemometric and machine-learning approaches primarily identify patterns associated with volatile organic compounds (VOCs), biogenic amines, organic acids, and other spoilage biomarkers detected by sensor arrays and electronic nose systems [353,354,355,356,357].

Among the most common approaches, multivariate statistical methods such as principal component analysis (PCA) and linear discriminant analysis (LDA) are widely used to visualize differences between fresh and spoiled products and classify samples by storage conditions or spoilage stage. Machine-learning algorithms, including support vector machines (SVMs) and artificial neural networks (ANNs), have further improved classification accuracy by identifying complex relationships between sensor responses and food quality parameters [358,359,360,361,362].

Integrating sensor technologies with machine learning has shown particular value in electronic nose and electronic tongue systems, where multiple sensor responses are analyzed simultaneously [363,364,365,366,367,368,369]. These approaches have been successfully applied to freshness assessment in meat and seafood products, monitoring fermentation and quality changes in dairy products, and evaluating aroma profiles in beverages such as wine and beer [282,370,371,372,373,374,375,376,377].

More recently, researchers have developed predictive models to estimate shelf life and forecast spoilage progression rather than simply classify product quality at a single time point [378]. Such approaches combine sensor-derived information with storage conditions and historical datasets to support real-time decision-making in food quality management systems [357]. Despite these advances, challenges related to sensor drift, data standardization, model transferability, and validation under industrial conditions remain important barriers to widespread implementation [378].

Artificial intelligence should be viewed as a complementary tool that enhances sensor data interpretation rather than as an independent monitoring technology. Its main contribution lies in improving the reliability, accuracy, and predictive capability of sensor-based food spoilage assessment systems.

Table 6 summarizes representative applications of chemometric and machine-learning approaches in sensor-based food spoilage monitoring.

## 8. Challenges and Limitations

Despite significant progress in developing chemical sensing technologies for food monitoring, several challenges still limit their widespread implementation in real-world industrial environments [122]. One main concern is the long-term stability of sensing materials, as many sensor platforms experience signal drift during prolonged operation due to surface contamination, aging of the sensing layer, or irreversible adsorption of analyte molecules [193,194]. These processes can alter baseline signals and sensitivity over time, compromising the reliability of quantitative measurements and requiring frequent recalibration procedures to maintain analytical accuracy [221]. Furthermore, environmental parameters such as temperature fluctuations, humidity levels, and oxygen concentration can influence adsorption–desorption dynamics at the sensor surface, introducing additional variability in sensor responses [122]. Beyond temporal stability, reproducibility across sensor batches remains a critical issue, as slight variations in nanomaterial synthesis, film deposition techniques, or device fabrication can significantly affect sensing performance [194].

Beyond stability-related challenges, the complex chemical composition of food matrices introduces additional limitations due to interference and insufficient selectivity [221]. Many sensing materials respond to broad classes of chemical compounds rather than specific target analytes, increasing the risk of cross-sensitivity when multiple volatile or non-volatile metabolites are present simultaneously [112]. This issue is particularly relevant in food systems where microbial metabolism generates diverse volatile organic compounds, including alcohols, aldehydes, organic acids, and sulfur-containing molecules that may produce overlapping sensor responses [221]. Although sensor arrays combined with chemometric or machine-learning approaches can partially mitigate these limitations by recognizing complex volatilomic patterns, environmental factors such as humidity and background gases can still affect classification-model accuracy [195].

Alongside analytical challenges, practical constraints related to scalability and economic feasibility also influence the industrial adoption of smart sensing technologies [221]. Many advanced sensors rely on nanostructured materials, functional polymers, or biological recognition elements whose fabrication and integration involve complex processes that may be difficult to scale for mass production [194]. Biosensor-based platforms often require immobilizing enzymes, antibodies, or aptamers, which can increase production costs and shorten device shelf life because these biological components are less stable [242]. Moreover, integrating sensing devices into intelligent packaging systems or wireless monitoring networks often requires additional electronic components for miniaturization, signal processing, and data transmission, further increasing system complexity and cost [122]. Consequently, achieving an optimal balance among analytical performance, manufacturability, and affordability remains critical to the successful commercialization of these technologies [289].

Finally, regulatory and standardization issues pose an additional barrier to the broader implementation of chemical sensors for food quality and safety monitoring [242]. Currently, the lack of harmonized protocols for sensor calibration, sampling procedures, and data interpretation limits comparability between studies and complicates regulatory acceptance of sensor-based measurements [122]. In applications involving direct contact with food or incorporation into packaging materials, sensing components must also comply with strict food safety regulations to prevent the migration of potentially hazardous substances into the product [195]. Therefore, validation against established analytical techniques, such as chromatographic or microbiological methods, is essential before sensor technologies can be reliably adopted for routine quality control or safety monitoring [221]. In addition, the increasing integration of sensing platforms into digital food monitoring systems poses further challenges regarding data standardization, traceability, and interoperability across complex supply chains [221]. Addressing these technological, economic, and regulatory limitations will be essential to transition smart chemical sensing systems from laboratory research to large-scale industrial applications [122].

From a technology-readiness perspective, colorimetric indicators, gas sensors, and certain electronic nose systems are among the most mature approaches and have already reached commercial or near-commercial deployment in intelligent packaging and food quality monitoring applications. In contrast, many biosensors, molecularly imprinted polymer sensors, and advanced nanomaterial-based platforms remain predominantly at the laboratory or pilot scale. Although these emerging technologies often provide superior sensitivity and selectivity, challenges related to long-term stability, large-scale manufacturing, cost, regulatory approval, and integration into food packaging systems continue to limit their commercial adoption. Future developments should therefore focus not only on improving analytical performance but also on scalability, robustness, and industrial validation under real operating conditions.

## 9. Future Perspectives

### 9.1. Wearable and Flexible Sensors

Real-time monitoring of food freshness is progressively shifting from laboratory-based analyses to flexible, low-cost sensing platforms, including wearable and package-integrated systems. These technologies enable continuous tracking of key spoilage indicators, such as gases, pH, and microbial activity, in both fermented and non-fermented foods, with the dual objective of reducing waste and enhancing food safety.

Among these approaches, flexible and package-integrated electronic systems have emerged as a central strategy for non-invasive, real-time quality assessment. For instance, paper-based electrical gas sensors (PEGS) incorporated into NFC-enabled smart packaging can detect ammonia (NH_3_) and hydrogen sulfide (H_2_S) released during spinach spoilage. These sensors operate wirelessly and can be interrogated by smartphones without requiring batteries [379]. Similarly, a miniaturized (2 × 2 cm) wireless spoilage sensor embedded in meat packaging detects biogenic amines using a functional polymer layer, with results accessible via mobile devices [380].

Building on these developments, flexible in situ optical systems (FIOS) integrate VIS/NIR spectral sensors onto bendable circuits that are wirelessly powered and cloud-connected, enabling real-time prediction of food quality directly at the product surface [381]. More broadly, recent reviews emphasize the rapid expansion of flexible physical, chemical, and biosensors based on materials such as carbon nanomaterials, conductive polymers, metal oxide semiconductors (MOXs), and hydrogels, all of which can conform to irregular food or packaging geometries [382].

Complementary to electronic sensing platforms, colorimetric and fluorometric systems constitute a widely explored class of flexible sensors for intelligent packaging. MOF–polymer films and hydrogels based on cellulose acetate or tragacanth enable detection of ammonia emissions from meat via visible color changes or fluorescence. These platforms are non-destructive, portable, and stable over several days, with straightforward readout via smartphone imaging [383,384]. Similarly, gelatin-based microneedle patches incorporating natural anthocyanins can be inserted into sealed fish packaging, where they respond to spoilage-related pH changes by producing quantifiable color changes, enabling consumer-level monitoring [385]. In parallel, researchers have widely investigated paper- and biopolymer-based indicator films, typically designed to be pH-responsive, as sustainable, biodegradable solutions for intelligent packaging applications [386,387,388].

In addition to optical and colorimetric approaches, e-nose technologies and gas microsensors play a crucial role in detecting volatile compounds associated with spoilage. Portable bioelectronic noses employing graphene-based field-effect transistors (FETs) functionalized with olfactory receptors achieve extremely low detection limits for biogenic amines in both liquid and gas phases [389]. Likewise, organic field-effect transistor (OFET)-based portable e-noses can discriminate among different food types (e.g., pork, chicken, fish, and milk) and assess freshness by detecting spoilage-related gases, with sensitivities below established safety thresholds [390].

Building on these capabilities, IoT-enabled e-nose systems incorporating multiple gas sensors (e.g., CO_2_, NH_3_, ethylene) enable continuous, networked monitoring of beef spoilage by correlating volatile organic compound (VOC) profiles with microbial growth and pH evolution [391]. Reviews of MEMS-based gas sensors and flexible sensing platforms further highlight their advantages in terms of miniaturization, low power consumption, and compatibility with integration into smart packaging or distributed sensing networks [27,41,392].

**Figure 2:**
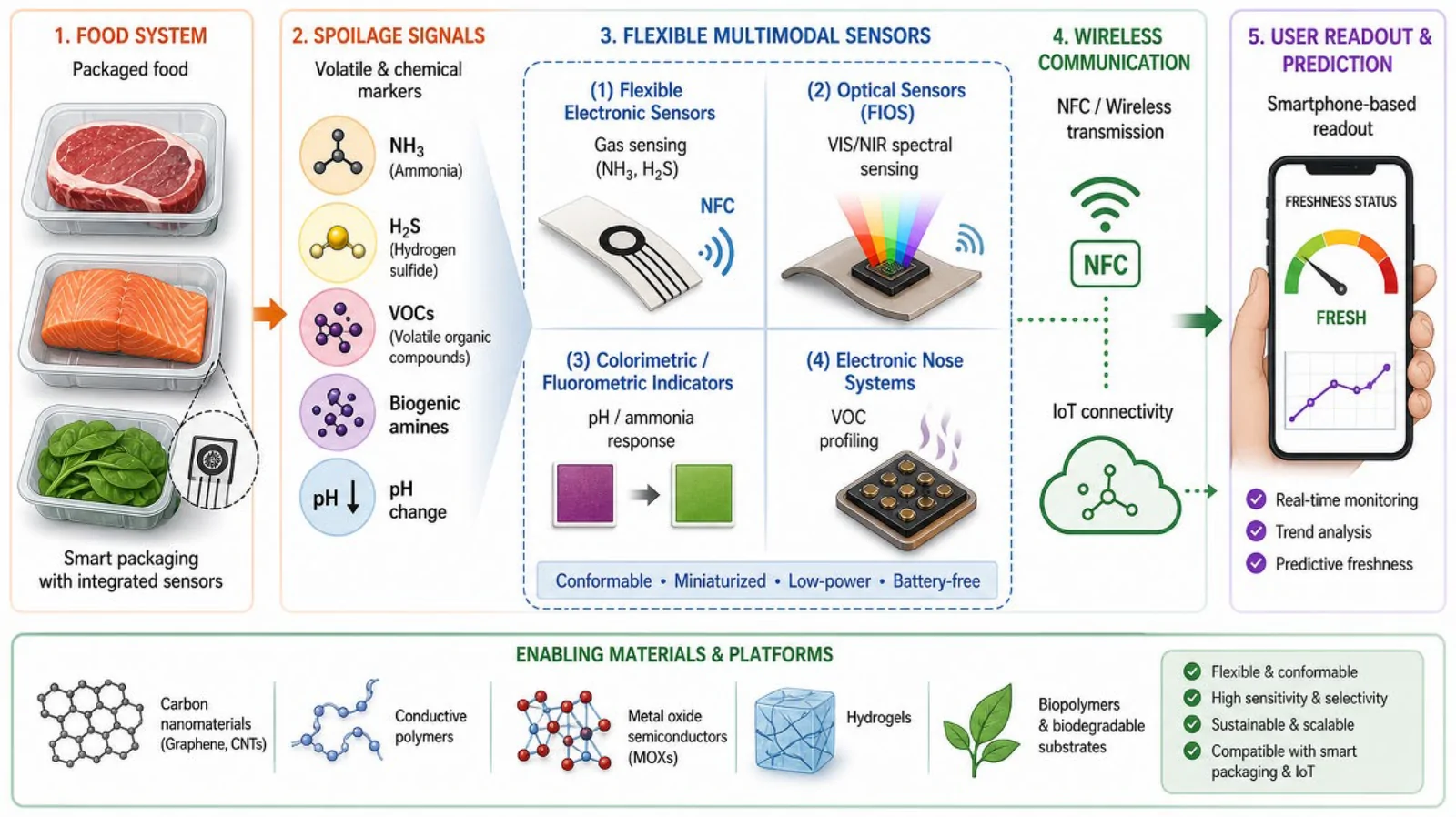
Flexible and package-integrated sensing systems enable non-invasive, real-time monitoring of food quality. Multimodal platforms detect spoilage-related chemical signals (e.g., NH_3_, H_2_S, VOCs, and pH changes), including electronic gas sensors, optical systems, colorimetric indicators, and electronic noses. These sensors integrate wirelessly via NFC and IoT technologies, enabling smartphone-based readout and predictive analysis of food freshness.

Beyond packaging-integrated systems, wearable and portable electrochemical platforms are emerging as versatile tools for on-site food analysis. Low-cost flexible electrochemical sensors, implemented in formats such as lab-on-a-glove devices, paper-based platforms, or leaf/disc substrates, have been proposed for in situ detection of food contaminants [393]. In particular, screen-printed electrode (SPE) sensors fabricated on flexible substrates enable direct detection of bacteria and biogenic amines at the point of need [27]. More broadly, recent reviews anticipate the convergence of wearable biosensors with IoT infrastructures, enabling real-time monitoring of fermentation processes, spoilage progression, and pathogen dynamics across diverse food categories, including dairy, meat, and fresh produce [389,394,395] (Figure 2).

In terms of application domains, current implementations of flexible, package-integrated, and wireless sensors predominantly focus on non-fermented foods, including fresh meat, fish, spinach, and milk [379,380,381,383,384,385,388,390,391]. In contrast, fermented foods are more commonly analyzed by advanced flavor-sensing techniques, such as electronic noses and tongues, gas chromatography–ion mobility spectrometry (GC-IMS), and nuclear magnetic resonance (NMR), which enable monitoring of fermentation dynamics, ripening, shelf life, and microbial activity based on volatile compound profiling [394]. Nevertheless, recent reviews highlight a growing shift toward real-time fermentation monitoring using IoT-enabled systems and, potentially, wearable sensing nodes [379,394,395]. Despite this progress, detailed device-level applications of flexible sensors in fermented matrices remain comparatively underexplored, even though many gas- and pH-based strategies developed for fresh foods are technically transferable.

From a mechanistic perspective, these sensing platforms rely on diverse transduction principles. These include variations in electrical conductivity (e.g., paper-based gas sensors, OFETs), changes in optical absorption or fluorescence (e.g., anthocyanins and natural dyes in films and hydrogels), and electrochemical signals such as current or potential (e.g., SPE-based sensors) [27,41,381,383,384,385,386,390].

In parallel, readout strategies are increasingly designed to enhance usability and accessibility. These include NFC-based smartphone interfaces, mobile applications for quantifying colorimetric and fluorometric responses, wireless cloud connectivity, and IoT-based dashboards for remote monitoring [380,381]. Collectively, these features enable non-destructive, real-time, or continuous monitoring of the food supply chain, from production through storage, often using low-cost and biodegradable materials [41,382,386,387,388].

Overall, flexible and wearable sensor platforms offer significant advantages, including high sensitivity (down to ppb or ppt levels), miniaturization, mechanical flexibility, conformability to food and packaging surfaces, and compatibility with low-cost, disposable manufacturing. Their integration with wireless communication and smartphone-based readout further enhances accessibility for both industrial and consumer applications [380,383,384].

However, key challenges persist, including the need for standardization, robust calibration across diverse food matrices, long-term operational stability, regulatory approval, and seamless integration into large-scale packaging and distribution systems [27,41,379,386,387,388,395].

Looking forward, the field is expected to evolve toward fully integrated, intelligent sensing ecosystems that combine flexible and wearable sensors with artificial intelligence and IoT infrastructures. Such systems hold strong potential for predictive spoilage modeling, real-time tracking of microbial dynamics, and expanded applicability to complex food systems, including fermented products [394,395].

### 9.2. Sustainable and Biodegradable Sensor Materials

**Figure 3:**
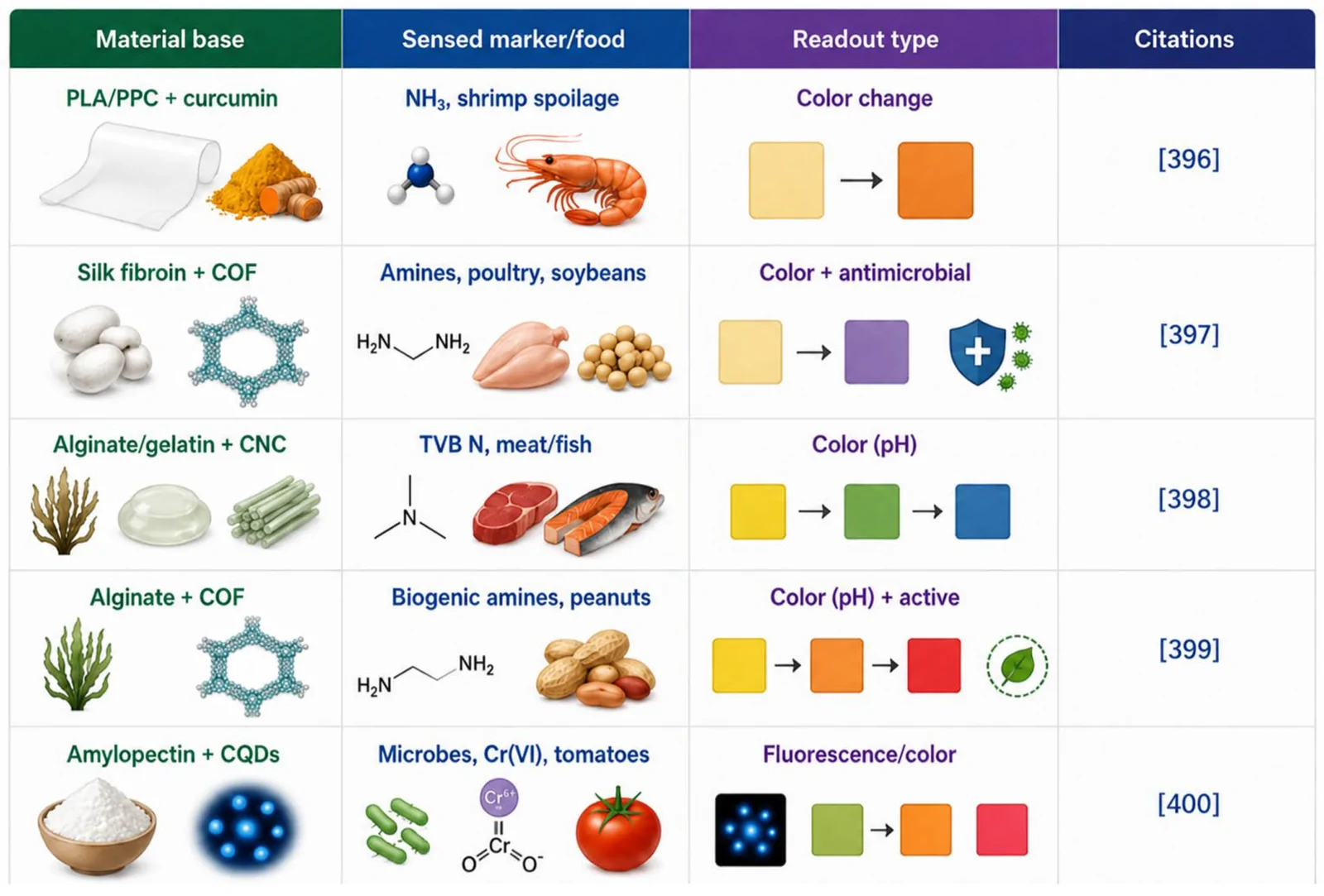
Illustrative overview of recent bio-based intelligent packaging systems for food quality monitoring. Adapted from [396,397,398,399,400].

Sustainable smart packaging aims to both protect food and signal when it is spoiling, reducing waste and plastic use. Recent research focuses on biodegradable polymers, natural dyes, and paper-based or hydrogel sensors that visually or electronically report spoilage gases, pH, or microbes. The main biodegradable sensor materials usually used are biopolymer films and hydrogels, such as (i) PLA/PPC films with curcumin that act as ammonia and shrimp spoilage indicators, turning from yellow to orange/red, while remaining largely bio-sourced and biodegradable [396]; (ii) Silk fibroin–COF membranes which show rapid (≈1 s) color change to poultry spoilage gases and can carry antimicrobial hexanal to slow mold in soybeans [397]; (iii) 3D-printed alginate/gelatin + nanocellulose hydrogels that stabilize pH dyes for TVB-N (meat/fish spoilage) and show clear color changes over several days [398]; (iv) Sodium alginate/COF strips pH-responsive which detect biogenic amines from meat, and can be loaded with citral for antimicrobial action [399]; (v) Amylopectin xerogels with onion-derived carbon quantum dots. They exhibit antimicrobial activity and show fluorescence/color changes in response to microbes and pH shifts associated with tomato spoilage [400]. Figure 3 summarizes the most representative biodegradable sensor matrices and spoilage markers. The figure summarizes representative material platforms, their target analytes or food matrices, and corresponding sensing readouts. PLA/PPC films incorporating curcumin detect ammonia associated with shrimp spoilage through visible color changes. Silk fibroin–COF composites respond to amines in poultry and soybeans and exhibit antimicrobial activity. Alginate/gelatin matrices reinforced with cellulose nanocrystals (CNCs) enable pH-based colorimetric detection of total volatile basic nitrogen (TVB-N) in meat and fish products. Alginate–COF systems detect biogenic amines in peanuts through active pH-responsive color changes, whereas amylopectin films embedded with carbon quantum dots (CQDs) provide fluorescence and colorimetric responses for monitoring microbial contamination, Cr(VI), and tomato quality. The examples highlight the versatility of sustainable biopolymer-based sensors for real-time food freshness assessment and safety monitoring.

Paper and cellulose are highlighted as low-cost, bio-based sensor matrices for gas/pH indicators [386,388]. As several authors note, laser-induced graphene on paper enables biodegradable, wireless gas/temperature sensing for real-time spoilage monitoring with smartphone readout [401]. Naik et al. [379] also note that paper-based electrical gas sensors in NFC-enabled labels monitor spoilage gases (e.g., NH_3_, H_2_S) in spinach through packaging in a battery-free manner. However, most demonstrations focus on non-fermented, high-protein, or fresh produce: meat, fish, poultry, shrimp, peanuts, soybeans, spinach, and tomatoes [379,396,397,398,399,400,401]. Nevertheless, reviews note that sensors that track pH changes, biogenic amines, CO_2_, TVB-N, and microbial activity across meat, seafood, fruits, and vegetables can, in principle, be extended to fermented foods, where these markers also change [386,388].

#### Final Remarks

Smart chemical sensors are transforming the way food freshness and spoilage are monitored, offering rapid, non-destructive, and increasingly real-time alternatives to conventional analytical approaches. As highlighted throughout this review, substantial progress has been made in developing electrochemical, optical, chemiresistive, gas-sensing, and biosensing platforms that can detect a broad range of spoilage indicators, including volatile organic compounds, biogenic amines, pH changes, and microbial metabolites. Advances in nanomaterials, functional polymers, and biorecognition elements have significantly improved sensor sensitivity, selectivity, response time, and adaptability to diverse food matrices.

These technologies have been demonstrated in both non-fermented and fermented foods, including meat, fish, dairy products, fruits, vegetables, beverages, and traditional fermented products. While most applications have focused on highly perishable foods, growing evidence indicates that smart sensing systems can also provide valuable information on fermentation dynamics, product maturation, flavor development, and deviations from desirable microbial pathways. This broader applicability highlights their potential not only as spoilage indicators but also as tools for process control and quality assurance.

The convergence of smart sensors with flexible electronics, intelligent packaging, wireless communication technologies, and artificial intelligence is further accelerating the transition from laboratory prototypes to connected food-monitoring ecosystems. Integration with RFID, NFC, IoT infrastructures, and machine-learning algorithms enables continuous data acquisition, remote monitoring, predictive shelf-life estimation, and improved traceability throughout the food supply chain. Such developments could enhance food safety, strengthen consumer confidence, optimize logistics, and help reduce global food loss and waste.

Despite these advances, important challenges remain before widespread commercial adoption. Sensor drift, limited selectivity in complex food matrices, environmental interferences, long-term operational stability, reproducibility, scalable manufacturing processes, and safety assessment of emerging sensing materials still require attention. In addition, the absence of harmonized validation protocols and regulatory frameworks limits comparability among studies and slows industrial implementation. Future research should therefore prioritize developing robust, standardized sensing platforms; validating against established analytical methods; using sustainable, food-safe materials; and developing cost-effective manufacturing strategies suitable for large-scale deployment.

Overall, smart chemical sensors are poised to become integral components of next-generation food quality management systems. Continued interdisciplinary collaboration among food scientists, chemists, materials scientists, engineers, data scientists, regulatory agencies, and industry stakeholders will be essential to translate these promising technologies into practical, reliable, and accessible solutions that support safer, more sustainable, and more resilient food systems.

Despite this progress, several important challenges continue to limit the widespread industrial adoption of smart chemical sensors. Sensor drift, limited selectivity in chemically complex food matrices, sensitivity to environmental variables such as temperature and humidity, and the lack of standardized validation protocols remain significant barriers to reliable deployment. In addition, many promising sensing systems are still evaluated under controlled laboratory conditions, with comparatively few studies demonstrating long-term performance in real food supply chains. Regulatory approval, manufacturing scalability, economic viability, and the development of food-safe and environmentally sustainable sensing materials also require further attention before large-scale commercialization.

Looking ahead, future research should move beyond optimizing individual sensors toward developing integrated, multimodal monitoring platforms that simultaneously track multiple spoilage indicators across diverse food matrices. Particular emphasis should be placed on integrating chemical sensing, intelligent packaging, wireless communication technologies, and artificial intelligence to enable predictive, rather than reactive, food quality management. Greater effort is also needed to establish standardized performance metrics, benchmark datasets, and validation protocols that enable comparison among sensing platforms and accelerate regulatory acceptance. Ultimately, the convergence of advanced materials, flexible electronics, machine learning, and IoT-based infrastructures is expected to drive the next generation of smart food-monitoring systems, transforming spoilage assessment from a point measurement into a continuous, data-driven, and predictive process across the entire food supply chain.

## Figures and Tables

**Table 1:** Key Chemical Markers of Spoilage.

Category	Examples	Origin/Formation	Significance in Spoilage Detection	References
Volatile Organic Compounds (VOCs)	Alcohols, aldehydes, ketones, esters	Microbial metabolism and lipid oxidation	Indicators of odor changes and overall spoilage	[57,58,59,69]
Biogenic Amines	Histamine, putrescine, cadaverine	Decarboxylation of amino acids by microorganisms	Indicators of protein degradation and potential toxicity	[61,62,65]
Organic Acids	Lactic acid, acetic acid, butyric acid	Carbohydrate fermentation by bacteria	Because a pH decrease indicates microbial activity	[51,70]
Gases	CO_2_, NH_3_, H_2_S	Microbial respiration and protein degradation	CO_2_ indicates microbial growth; NH_3_ and H_2_S indicate advanced spoilage	[52,53,57]

**Table 2:** Representative performance characteristics of major smart chemical sensor platforms used for food spoilage monitoring.

Sensor Type	Target Analyte(s)	Representative Food Matrix	Typical Analytical Performance	Main Advantages	Main Limitations	References
Metal oxide semiconductor (MOS) sensors	NH_3_, H_2_S, TMA, VOCs	Meat, fish, seafood	High sensitivity (typically in the ppb–ppm range) and rapid response (seconds to minutes)	Low cost, easy miniaturization, and compatibility with electronic nose systems	Humidity interference, sensor drift, limited selectivity	[107,108,109,110]
Conducting polymer sensors	Amines, VOCs	Meat, dairy products	Fast response and good sensitivity toward volatile spoilage compounds	Low operating temperature, flexible device integration	Lower long-term stability and environmental sensitivity	[82,111,112]
Carbon-based chemiresistive sensors (graphene, CNTs)	NH_3_, H_2_S, biogenic amines	Meat, fish, dairy products	High sensitivity due to large surface area and excellent electron transport properties	High conductivity, rapid signal transduction, and potential for flexible devices	Reproducibility and surface-functionalization challenges	[43,113,114,115]
Electrochemical sensors	Biogenic amines, organic acids, microbial metabolites	Meat, dairy, processed foods	Low detection limits (typically μM–nM range) and rapid quantitative analysis	High sensitivity, quantitative outputs, suitability for portable devices	Electrode fouling and calibration requirements	[78,89]
Optical/colorimetric sensors	pH changes, volatile amines, CO_2_	Fish, meat, intelligent packaging systems	Visual detection of spoilage-related changes within minutes	Low-cost, non-destructive analysis, direct interpretation by consumers	Usually semi-quantitative; affected by storage and lighting conditions	[92,116,117]
Fluorescence sensors	VOCs, biogenic amines	Meat, fish, cheese	Very high sensitivity, commonly reported in the nM–μM range	Excellent sensitivity and tunable selectivity	Requires optical instrumentation and signal processing	[93,94]
Biosensors (enzyme-, antibody-, or aptamer-based)	Histamine, tyramine, pathogens, toxins	Fish, fermented foods, dairy products	High selectivity and low detection limits (typically nM–μM range)	Target-specific recognition and high analytical accuracy	Stability and cost of biological recognition elements	[99,118]
Molecularly imprinted polymer (MIP) sensors	Histamine, tyramine, biogenic amines	Cheese, fermented meat products	High selectivity and low μM detection levels	Good chemical stability and reusability	More complex fabrication procedures	[119,120]
Electronic nose (e-nose) systems	VOC patterns rather than individual analytes	Meat, fish, dairy products, beverages	Real-time pattern recognition and classification of volatile profiles	Simultaneous detection of multiple spoilage markers	Requires chemometric and machine-learning models	[69,121,122]
Electronic tongue (e-tongue) systems	Organic acids, dissolved metabolites, taste-active compounds	Wine, beer, yogurt, and other liquid foods	Multivariate analysis of non-volatile compounds in real time	Complements e-nose systems and is suitable for liquid matrices	Matrix effects and frequent recalibration requirements	[123,124]

**Table 4:** Sensor Mechanisms Based on Microbial Metabolite Detection for Spoilage.

Sensor Type	Detection Mechanism	Target Microbial Metabolites	Working Principle	Typical Applications	References
Gas Sensors (MOS, semiconductor)	Volatile compound detection	NH_3_, H_2_S, CO_2_, TMA, VOCs	Change in electrical resistance due to the adsorption and reaction of volatile gases on the sensor surface.	Meat, fish, and poultry spoilage monitoring	[208]
Electrochemical Sensors	Redox reaction detection	H_2_O_2_, amines, organic acids	Measurement of current/voltage changes due to oxidation-reduction reactions	Meat, dairy, processed foods	[78]
Optical Sensors (Fluorescence/Absorbance)	Light-based detection	VOCs, CO_2_, microbial by-products	Changes in absorbance/fluorescence signals due to chemical interactions	Packaged foods, freshness indicators	[209]
CO_2_ Sensors (Infrared-based)	Gas concentration measurement	CO_2_ (respiration & microbial growth)	Infrared absorption is proportional to CO_2_ concentration	Fruits, vegetables, and packaged foods	[210]

**Table 5:** Applications of electronic noses in enology are organized by sensor technology and data analysis approaches. Adapted from Alfieri et al. [262].

Sensor Type	Chemometric Approach	Typical Applications	Reference Techniques	References
MOS	PCA	Wine aging, aroma profiling, and defect detection	GC-MS, Wet chemistry	[264,265,267,268,269,270]
MOS	ML/SVM/DL	Defect classification, wine identification, fault detection	–/Wet chemistry	[259,261,270,271,272]
MOS	PLS/PLS-DA	Quantification and classification of wines	GC-MS, Sensory analysis	[267,273]
QMB	PCA	Detection of spoilage, grape maturity, and winemaking effects	GC-MS, Wet chemistry	[266,274,275]
QMB	PLS-DA	Sparkling wine aging, noble rot detection	GC-FID, Wet chemistry	[268,276]
CP/SAW	Statistical methods	Fruit identification and aroma characterization	GC-MS, Wet chemistry	[277,278]
Hybrid (MOS-QMB, others)	LDA/ANN/ELM	Wine classification and authentication	Wet chemistry, Sensory	[279]

Metal Oxide Semiconductors—MOS; Quartz Microbalance—QMB; conduction polymer—CP; surface acoustic wave—SAW; Principal Component Analysis—PCA; Machine Learning—ML; Support Vector Machines—SVM; Deep Learning—DL; PLS—Partial Least Squares; PLS-DA—Partial Least Squares Discriminant Analysis; Linear Discriminant Analysis—LDA; Artificial Neural Networks—ANN; Extreme Learning Machines—ELM; Gas chromatography-mass spectrometry—GC-MS; Gas Chromatography Flame Ionization Detection—GC-FID.

**Table 6:** Chronological summary of key experimental studies that have shaped the current landscape of AI-driven food quality monitoring.

Matrix	Sensor Technology	AI/Analytical Approach	Primary Outcome	Reference
Seafood	Optical/Vision-based	CNN-Attention	Detecting ammonia in complex backgrounds.	[371]
Douro Wine	E-Sensors/Panel	Chemometrics/PCA	Decoding terroir influence via electronic analysis.	[272]
Packaged Food	pH-sensitive Labels	LSTM [Deep Learning]	96% accuracy in shelf-life [RUL] prediction.	[370]
Fish	Gas Array	CNN-Transfer Learning	Accelerating spoilage detection via pre-trained models.	[73]
Seafood	Optical/Camera	ResNet-50	Image-based spoilage detection via skin color.	[372]
Beef	Hyper-spectral	CNN/PLS	Prediction of microbial counts via spectral data.	[373]
Fresh Produce	Chemiresistive Nano	Random Forest	Drift-resilient monitoring of spoilage VOCs.	[301]
Fruits/Veg	IoT-E-nose	LDA/ANN	Development of low-cost portable monitoring nodes.	[375]
Vegetables	Multi-sensor IoT	Edge AI/ANN	Real-time spoilage alerts at the container level.	[376]
Yogurt/Dairy	E-nose + E-tongue	SVM/PCA	Identification of abnormal fermentation profiles.	[233]
Fruit/Peach	E-nose + NIR	ANN/PLS Data Fusion	Enhanced accuracy via synergistic sensing.	[377]
Post-harvest	MOS E-nose	PLS-DA	Real-time tracking of grape metabolic decay.	[368]
Fish/Seafood	Electrochemical Array	MLP-ANN	Rapid in situ detection of biogenic amines.	[361]
Cheese	Potentiometric Tongue	PLS Regression	Quantitative monitoring of biogenic amines.	[370]
Pork Meat	E-nose [Visualized]	Deep Belief Networks	Feature extraction from gas-sensing images.	[374]
Beef/Meat	MOX Sensor Array	XGBoost/k-NN	Classification of beef freshness via IoT cloud.	[362]

## Data Availability

No new data were created or analyzed in this study. Data sharing is not applicable.

## Abbreviations

The following abbreviations are used in this manuscript:

AI	Artificial intelligence
ANN	Artificial neural network
AgNPs	Silver nanoparticles
AuNPs	Gold nanoparticles
CNNs	Convolutional neural networks
CNTs	Carbon nanotubes
COF	Covalent organic framework
DL	Deep learning
FETs	Graphene-based field-effect transistors
FIOS	Flexible in situ optical systems
FTIR	Fourier transform infrared spectroscopy
GCE	Glass carbon electrode
GC-IMS	Gas chromatography-ion mobility spectrometry
GC–MS	Gas chromatography–mass spectrometry
HPLC	High-performance liquid chromatography
IMS	Ion mobility spectrometry
LDA	Linear discriminant analysis
LSTM	Long Short-Term Memory
LSPR	Localized surface plasmon resonance
MAP	Modified atmosphere packaging
MEMS	Microelectromechanical systems
MIPs	Molecularly imprinted polymers
MOFs	Metal–organic frameworks
MOS	Metal oxide semiconductor
MVOCs	Microbial volatile organic compounds
MWCNTs	Modified with multi-walled carbon nanotubes
NDTTs	Non-destructive testing techniques
NMR	Nuclear magnetic resonance
OFET	Organic field-effect transistor
PCA	Principal component analysis
PEGS	Paper-based electrical gas sensors
PLA	Poly(lactic acid)
PLS	Partial least squares
PPC	Poly(propylene) carbonate
QCM	Quartz crystal microbalance
RF	Random Forest
RNN	Recurrent Neural Networks
RUL	Remaining Useful Life
SAW	Surface acoustic wave
SPE	Screen-printed electrode
SVM	Support Vector Machine
TMA	Trimethylamine
TYR	Tyrosinase
VOCs	Volatile organic compounds

## References

[1] Steele R. (2004). Understanding and Measuring the Shelf-Life of Food.

[2] Singh R.P., Anderson B.A., Steele R. (2004). The major types of food spoilage: An overview. Understanding and Measuring the Shelf-Life of Food.

[3] Roudaut G., Debeaufort F., Skibsted L.H., Risbo J., Andersen M.L. (2010). Moisture loss, gain and migration in foods and its impact on food quality. Chemical Deterioration and Physical Instability of Food and Beverages.

[4] Troller J.A. (2012). Water Activity and Food.

[5] Roos Y., Karel M. (1991). Plasticizing effect of water on thermal behavior and crystallization of amorphous food models. J. Food Sci..

[6] Karanth S., Feng S., Patra D., Pradhan A.K. (2023). Linking microbial contamination to food spoilage and food waste: The role of smart packaging, spoilage risk assessments, and date labeling. Front. Microbiol..

[7] Mafe A.N., Edo G.I., Makia R.S., Joshua O.A., Akpoghelie P.O., Gaaz T.S., Jikah A.N., Yousif E., Isoje E.F., Igbuku U.A. (2024). A review on food spoilage mechanisms, foodborne diseases and commercial aspects of food preservation and processing. Food Chem. Adv..

[8] Rogers L.D., Overall C.M. (2013). Proteolytic post-translational modification of proteins: Proteomic tools and methodology. Mol. Cell. Proteom..

[9] Zhang K., Zhang T.T., Guo R.R., Ye Q., Zhao H.L., Huang X.H. (2023). The regulation of key flavors of traditional fermented food by microbial metabolism: A review. Food Chem. X.

[10] Chandra P., Enespa, Singh R., Arora P.K. (2020). Microbial lipases and their industrial applications: A comprehensive review. Microb. Cell Fact..

[11] Park S.H., Na Y., Kim J., Kang S.D., Park K.H. (2018). Properties and applications of starch modifying enzymes for use in the baking industry. Food Sci. Biotechnol..

[12] Horstmann S.W., Lynch K.M., Arendt E.K. (2017). Starch characteristics linked to gluten-free products. Foods.

[13] Moon K.M., Kwon E.B., Lee B., Kim C.Y. (2020). Recent trends in controlling the enzymatic browning of fruit and vegetable products. Molecules.

[14] Kathuria D., Gautam S., Thakur A. (2023). Maillard reaction in different food products: Effect on product quality, human health and mitigation strategies. Food Control.

[15] Petruzzi L., Corbo M.R., Sinigaglia M., Bevilacqua A. (2017). Microbial spoilage of foods: Fundamentals. The Microbiological Quality of Food.

[16] Veld J.H.H. (1996). Microbial and biochemical spoilage of foods: An overview. Int. J. Food Microbiol..

[17] Amit S.K., Uddin M.M., Rahman R., Islam S.M.R., Khan M.S. (2017). A review on mechanisms and commercial aspects of food preservation and processing. Agric. Food Secur..

[18] Sun X., Wang J., Dong M., Zhang H., Li L., Wang L. (2022). Food spoilage, bioactive food fresh-keeping films and functional edible coatings: Research status, existing problems and development trend. Trends Food Sci. Technol..

[19] Chhetri K.B. (2024). Applications of artificial intelligence and machine learning in food quality control and safety assessment. Food Eng. Rev..

[20] Aung M.M., Chang Y.S. (2014). Temperature management for the quality assurance of a perishable food supply chain. Food Control.

[21] Yar M.S., Ibeogu I.H., Regmi A., Zhang N., Li C. (2025). Advances in intelligent time-temperature indicators for cold chain monitoring: Mechanisms, challenges, and applications. Trends Food Sci. Technol..

[22] da Costa T.P., Gillespie J., Cama-Moncunill X., Ward S., Condell J., Ramanathan R., Murphy F. (2023). A systematic review of real-time monitoring technologies and its potential application to reduce food loss and waste: Key elements of food supply chains and IoT technologies. Sustainability.

[23] Badia-Melis R., Mishra P., Ruiz-García L. (2015). Food traceability: New trends and recent advances. A review. Food Control.

[24] Misra N.N., Dixit Y., Al-Mallahi A., Bhullar M.S., Upadhyay R., Martynenko A. (2022). IoT, big data, and artificial intelligence in agriculture and food industry. IEEE Internet Things J..

[25] Ablegue A.G.M.-D., Zhang M., Guo Q., Rui L. (2025). Monitoring and Shelf Life Extension of Food Products Based on Machine Learning and Deep Learning: Progress and Potential Applications. Food Rev. Int..

[26] Abass T., Eruaga M.A., Itua E.O., Bature J.T. (2024). Advancing food safety through IoT: Real-time monitoring and control systems. Int. Med. Sci. Res. J..

[27] Torres-Sánchez R., Martínez-Zafra M.T., Castillejo N., Guillamón-Frutos A., Artés-Hernández F. (2020). Real-time monitoring system for shelf-life estimation of fruits and vegetables. Sensors.

[28] Ferone M., Gowen A., Fanning S., Scannell A.G.M. (2020). Microbial detection and identification methods: Bench top assays to omics approaches. Compr. Rev. Food Sci. Food Saf..

[29] Johnson N.A.N., Adade S.Y.S.S., Ekumah J.N., Kwadzokpui B.A., Xu J., Xu Y., Chen Q. (2025). A comprehensive review of analytical techniques for spice quality and safety assessment in the modern food industry. Crit. Rev. Food Sci. Nutr..

[30] Ma Y., Yang W., Xia Y., Xue W., Wu H., Li Z., Zhang F., Qiu B., Fu C. (2022). Properties and applications of intelligent packaging indicators for food spoilage. Membranes.

[31] Foddai A.C.G., Grant I.R. (2020). Methods for detection of viable foodborne pathogens: Current state-of-the-art and future prospects. Appl. Microbiol. Biotechnol..

[32] Merino N., Espina L., Berdejo D., Pagán R., García-Gonzalo D. (2026). Deciphering poultry microbial ecosystems by classical and modern tools. Food Biosci..

[33] Lytou A.E., Panagou E.Z., Nychas G.J.E. (2019). Volatilomics for food quality and authentication. Curr. Opin. Food Sci..

[34] Mayr D., Margesin R., Klingsbichel E., Hartungen E., Jenewein D., Schinner F., Märk T.D. (2003). Rapid detection of meat spoilage by measuring volatile organic compounds using proton transfer reaction mass spectrometry. Appl. Environ. Microbiol..

[35] Ellis D.I., Goodacre R., Blackburn C.W. (2006). Quantitative detection and identification methods for microbial spoilage. Food Spoilage Microorganisms.

[36] Fernández-No I.C., Calo-Mata P., Böhme K., Cepeda A., Calo-Mata P., Barros-Velázquez J. (2011). Detection and quantification of spoilage and pathogenic *Bacillus* species by real-time PCR. Food Microbiol..

[37] Adeyeye S.A.O., Adeyeye B.R. (2025). Recent Advances in Electronic Eye, Electronic Nose, and Electronic Tongue in Sensory Evaluation of Food: A Review. J. Food Process Eng..

[38] Abi-Rizk H., Bouveresse D.J.R., Chamberland J., Cordella C.B. (2023). Recent developments of e-sensing devices coupled to data processing techniques in food quality evaluation: A critical review. Anal. Methods.

[39] Arteaga-Cabrera E., Ramírez-Márquez C., Sánchez-Ramírez E., Segovia-Hernández J.G., Osorio-Mora O., Gómez-Salazar J.A. (2025). Advancing optimization strategies in the food industry: From traditional approaches to multi-objective and technology-integrated solutions. Appl. Sci..

[40] Mustafa F., Andreescu S. (2018). Chemical and biological sensors for food-quality monitoring and smart packaging. Foods.

[41] Mohammadi Z., Jafari S.M. (2020). Detection of food spoilage and adulteration by novel nanomaterial-based sensors. Adv. Colloid Interface Sci..

[42] Nanda P.K., Bhattacharya D., Das J.K., Bandyopadhyay S., Ekhlas D., Lorenzo J.M., Gagaoua M. (2022). Emerging role of biosensors and chemical indicators to monitor the quality and safety of meat and meat products. Chemosensors.

[43] Ghosh T., Raj G.B., Dash K.K. (2022). A comprehensive review on nanotechnology based sensors for monitoring quality and shelf life of food products. Meas. Food.

[44] Ben-Daya M., Hassini E., Bahroun Z., Banimfreg B.H. (2021). The role of internet of things in food supply chain quality management: A review. Qual. Manag. J..

[45] Shruti A., Bage N., Kar P. (2024). Nanomaterials based sensors for analysis of food safety. Food Chem..

[46] Ye Z., Liu Y., Li Q. (2021). Recent progress in smart electronic nose technologies enabled with machine learning methods. Sensors.

[47] Anwar H., Anwar T., Murtaza S. (2023). Review on food quality assessment using machine learning and electronic nose system. Biosens. Bioelectron. X.

[48] Poeta E., Liboà A., Mistrali S., Núñez-Carmona E., Sberveglieri V. (2023). Nanotechnology and e-sensing for food chain quality and safety. Sensors.

[49] Sukhavattanakul P. (2025). Sensing food spoilage with nanotechnology: A review of current research and challenges. Meas. Food.

[50] European Parliament and of the Council (2002). Regulation (EC) No 178/2002 of 28 January 2002 laying down the general principles and requirements of food law, establishing the European Food Safety Authority and laying down procedures in matters of food safety. Off. J. Eur. Communities.

[51] Dainty R.H. (1996). Chemical/biochemical detection of spoilage. Int. J. Food Microbiol..

[52] Gram L., Huss H.H. (1996). Microbiological spoilage of fish and fish products. Int. J. Food Microbiol..

[53] Lázaro C.A., Conte-Júnior C.A., Canto A.C., Monteiro M.L.G., Costa-Lima B., Cruz A.G., Mársico E.T., Franco R.M. (2015). Biogenic amines as bacterial quality indicators in different poultry meat species. LWT.

[54] Uhlig E., Bucher M., Strenger M., Kloß S., Schmid M. (2024). Towards reducing food wastage: Analysis of degradation products formed during meat spoilage under different conditions. Foods.

[55] Hempel A.W., O’Sullivan M.G., Papkovsky D.B., Kerry J.P. (2013). Use of smart packaging technologies for monitoring and extending the shelf-life quality of modified atmosphere packaged bread. Eur. Food Res. Technol..

[56] Preethichandra D.M.G., Gholami M.D., Izake E.L., O’Mullane A.P., Sonar P. (2023). Conducting polymer-based ammonia and hydrogen sulfide chemical sensors and their suitability for detecting food spoilage. Adv. Mater. Technol..

[57] Palanisamy Y., Kadirvel V., Ganesan N.D. (2025). Recent technological advances in food packaging: Sensors, automation, and application. Sustain. Food Technol..

[58] Ha Y. (2025). Advancements in gaseous sensor technology for ensuring food safety: A review. Int. J. Food Sci. Technol..

[59] Raza W., Ling N., Yang L., Huang Q., Shen Q. (2016). Response of tomato wilt pathogen *Ralstonia solanacearum* to volatile organic compounds produced by *Bacillus amyloliquefaciens* SQR-9. Sci. Rep..

[60] Quaglia M., Ederli L., Pasqualini S., Zazzerini A. (2011). Biological control agents and chemical inducers of resistance for postharvest control of *Penicillium expansum* on apple fruit. Postharvest Biol. Technol..

[61] Ruiz-Capillas C., Herrero A.M. (2019). Impact of biogenic amines on food quality and safety. Foods.

[62] Halász A., Baráth Á., Simon-Sarkadi L., Holzapfel W. (1994). Biogenic amines and their production by microorganisms in food. Trends Food Sci. Technol..

[63] Omer A.K., Mohammed R.R., Ameen P.S.M., Abas Z.A., Ekici K. (2021). Presence of biogenic amines in food and their public health implications: A review. J. Food Prot..

[64] Durak-Dados A., Michalski M., Osek J. (2020). Histamine and other biogenic amines in food. J. Vet. Res..

[65] Biji K.B., Ravishankar C.N., Venkateswarlu R., Mohan C.O., Gopal T.K.S. (2016). Biogenic amines in seafood: A review. J. Food Sci. Technol..

[66] Ding T., Li Y. (2024). Biogenic amines are important indices for characterizing the freshness and hygienic quality of aquatic products: A review. LWT.

[67] Mahmoudzadeh M., Abedi A.-S., Moslemi M., Pilevar Z., Ghani A. (2022). Biogenic amines in foods: Surveying effective factors and measuring methods. J. Microbiol. Biotechnol. Food Sci..

[68] Moniente M., Botello-Morte L., García-Gonzalo D., Pagán R., Ontañón I. (2022). Analytical strategies for the determination of biogenic amines in dairy products. Compr. Rev. Food Sci. Food Saf..

[69] Wilson A.D., Baietto M. (2009). Applications and advances in electronic-nose technologies. Sensors.

[70] Nychas G.-J.E., Skandamis P.N., Tassou C.C., Koutsoumanis K.P. (2008). Meat spoilage during distribution. Meat Sci..

[71] Nath S. (2024). Advancements in food quality monitoring: Integrating biosensors for precision detection. Sustain. Food Technol..

[72] Cheng Y., Wang K., Xu H., Li T., Jin Q., Cui D. (2021). Recent developments in sensors for wearable device applications. Anal. Bioanal. Chem..

[73] Chen X., Lu S., Zhang Y. (2024). Deep learning-based gas sensor arrays for food freshness monitoring: A review. Anal. Chim. Acta.

[74] He X., Pu Y., Chen L., Jiang H., Xu Y., Cao J., Jiang W. (2023). A comprehensive review of intelligent packaging for fruits and vegetables: Target responders, classification, applications, and future challenges. Compr. Rev. Food Sci. Food Saf..

[75] Uztürk D., Büyüközkan G. (2024). Industry 4.0 technologies in Smart Agriculture: A review and a Technology Assessment Model proposition. Technol. Forecast. Soc. Change.

[76] Dhar P., Bhowmik A., Nath P.C., Patel D., Shankar A., Tomar P., Sinha A. (2026). Smart Technologies in Food Safety and Quality Monitoring: A Review of Technological Advances, Opportunities and Challenges. J. Food Process Eng..

[77] Abdulhussain S.H., Mahmmod B.M., Alwhelat A., Shehada D., Shihab Z.I., Mohammed H.J., Abdulameer T.H., Alsabah M., Fadel M.H., Ali S.K. (2025). A Comprehensive Review of Sensor Technologies in IoT: Technical Aspects, Challenges, and Future Directions. Computers.

[78] Mishra G.K., Barfidokht A., Tehrani F., Mishra R.K. (2018). Food Safety Analysis Using Electrochemical Biosensors. Foods.

[79] Vergara A., Llobet E. (2012). Sensor selection and chemo-sensory optimization: Toward an adaptable chemo-sensory system. Front. Neuroeng..

[80] Borah H., Gogoi S., Kalita S., Puzari P. (2018). A Broad Spectrum Amperometric pesticide biosensor based on glutathione S-transferase immobilized on graphene oxide-gelatin matrix. J. Electroanal. Chem..

[81] Bucur B., Purcarea C., Andreescu S., Vasilescu A. (2021). Addressing the selectivity of enzyme biosensors: Solutions and perspectives. Sensors.

[82] Vallejos S., Trigo-López M., Arnaiz A., Miguel Á., Muñoz A., Mendía A., García J.M. (2022). From Classical to Advanced Use of Polymers in Food and Beverage Applications. Polymers.

[83] Umapathi R., Park B., Sonwal S., Rani G.M., Cho Y., Huh Y.S. (2022). Advances in optical-sensing strategies for the on-site detection of pesticides in agricultural foods. Trends Food Sci. Technol..

[84] Zhao M., Wang Y., Jin R., Li H., Su X., Yan X. (2026). Advances in Optical Biosensors for Pesticide Detection. Research.

[85] Kaur M., Gaba J., Singh K., Bhatia Y., Singh A., Singh N. (2023). Recent Advances in Recognition Receptors for Electrochemical Biosensing of Mycotoxins-A Review. Biosensors.

[86] Zhang L., Zhang J., Cui M., Qi W., Huang R., Su R., Zhang K. (2026). Multifunctional Bio-Based Packaging for Perishable Foods: Structural Design, Scalable Fabrication, and Versatile Applications. Adv. Mater..

[87] Tarlak F. (2023). The Use of Predictive Microbiology for the Prediction of the Shelf Life of Food Products. Foods.

[88] Baranwal J., Barse B., Gatto G., Broncova G., Kumar A. (2022). Electrochemical Sensors and Their Applications: A Review. Chemosensors.

[89] Del Valle M. (2010). Electronic tongues employing electrochemical sensors. Electroanalysis.

[90] Silveri F., Della Pelle D., Compagnone D. (2025). Recent advances in sustainable strategies for the integration of nanostructured sensing surfaces in electroanalytical devices. Trends Anal. Chem..

[91] Wang Y., Yang Y., Liu H. (2026). A Review of High-Throughput Optical Sensors for Food Detection Based on Machine Learning. Foods.

[92] Yu D., Cheng S., Li Y., Su W., Tan M. (2024). Recent advances on natural colorants-based intelligent colorimetric food freshness indicators: Fabrication, multifunctional applications and optimization strategies. Crit. Rev. Food Sci. Nutr..

[93] Zhou J., Gui Y., Lv X., He J., Xie F., Li J., Cai J. (2022). Nanomaterial-Based Fluorescent Biosensor for Food Safety Analysis. Biosensors.

[94] Deshpande K., Kanungo L. (2023). Chemiluminescence and fluorescence biosensors for food application: A review. Sens. Actuators Rep..

[95] Jayan H., Zhou R., Sun C., Wang C., Yin L., Zou X., Guo Z. (2025). Intelligent Gas Sensors for Food Safety and Quality Monitoring: Advances, Applications, and Future Directions. Foods.

[96] Andre R.S., Mercante L.A., Facure M.H.M., Sanfelice R.C., Fugikawa-Santos L., Swager T.M., Correa D.S. (2022). Recent progress in amine gas sensors for food quality monitoring: Novel architectures for sensing materials and systems. ACS Sens..

[97] Singh P., Habiba U., Shafi Z., Noor A., Pandey V.K., RahulSingh R. (2025). Understanding the concepts of smart e-nose technology in combination with machine learning for new era of food safety: An advanced review. Food Saf. Health.

[98] Inês A., Cosme F. (2025). Biosensors for Detecting Food Contaminants—An Overview. Processes.

[99] Cavalcante F.T.T., Falcão I.R.d.A., Souza J.E.d.S., Rocha T.G., de Sousa I.G., Cavalcante A.L.G., de Oliveira A.L.B., de Sousa M.C.M., dos Santos J.C.S. (2021). Designing of Nanomaterials-Based Enzymatic Biosensors: Synthesis, Properties, and Applications. Electrochem.

[100] Garrido-Momparler V., Peris M. (2022). Smart sensors in environmental/water quality monitoring using IoT and cloud services. Trends Environ. Anal. Chem..

[101] Sobhan A., Hossain A., Wei L., Muthukumarappan K., Ahmed M. (2025). IoT-Enabled Biosensors in Food Packaging: A Breakthrough in Food Safety for Monitoring Risks in Real Time. Foods.

[102] Villegas-Ch W., Gutiérrez R., Govea J., García-Ortiz J. (2025). Integrated AI, IoT, and blockchain for enhancing security and traceability in perishable logistics. Emerg. Sci. J..

[103] Alarood A., Ibrahim A., Abubakar A., Alsulami A. (2025). Examining the factors influencing IoT-blockchain-based secure transmission services. Sci. Rep..

[104] Li X., Xu C., Sun J., Long Y., Wang Y., Li Z., Bai C., Du X. (2024). Room-temperature agricultural ethylene detection by freestanding three-dimensional porous-ZnO/carbon nanofibers. Sens. Actuators B Chem..

[105] Lawaniya S.D., Awasthi A., Menezes P.W., Awasthi K. (2025). Detection of foodborne pathogens through volatile organic compounds sensing via metal oxide gas sensors. Adv. Sens. Res..

[106] Kuswandi B., Hidayat M.A., Noviana E. (2022). Paper-based electrochemical biosensors for food safety analysis. Biosensors.

[107] Berna A. (2010). Metal oxide sensors for electronic noses and their application to food analysis. Sensors.

[108] Galstyan V., Bhandari M.P., Sberveglieri V., Sberveglieri G., Comini E. (2018). Metal oxide nanostructures in food applications: Quality control and packaging. Chemosensors.

[109] Wusiman M., Taghipour F. (2022). Methods and mechanisms of gas sensor selectivity. Crit. Rev. Solid State Mater. Sci..

[110] Wu K., Debliquy M., Zhang C. (2023). Metal–oxide–semiconductor resistive gas sensors for fish freshness detection. Compr. Rev. Food Sci. Food Saf..

[111] Arshak K., Moore E., Lyons G.M., Harris J., Clifford S. (2004). A review of gas sensors employed in electronic nose applications. Sens. Rev..

[112] Li Z., Suslick K.S. (2021). The optoelectronic nose: Colorimetric and fluorometric sensor arrays. Chem. Rev..

[113] Varghese S.S., Varghese S.H., Swaminathan S., Singh K.K., Mittal V. (2015). Two-dimensional materials for sensing: Graphene and beyond. Electronics.

[114] Yáñez-Sedeño P., Pingarrón J.M., Riu J., Rius F.X. (2010). Electrochemical sensing based on carbon nanotubes. TrAC Trends Anal. Chem..

[115] Guo S., Radecka I., Eissa A.M., Ivanov E., Stoeva Z., Tchuenbou-Magaia F. (2025). Recent advances in carbon-based sensors for food and medical packaging under transit: A focus on humidity, temperature, mechanical, and multifunctional sensing technologies—A systematic review. Materials.

[116] Kuswandi B., Wicaksono Y., Jayus, Abdullah A., Heng L.Y., Ahmad M. (2011). Smart packaging: Sensors for monitoring food quality and safety. Sens. Instrum. Food Qual. Saf..

[117] Silva-Pereira M.C., Teixeira J.A., Pereira-Júnior V.A., Stefani R. (2015). Chitosan/corn starch blend films with extract from *Brassica oleracea* (red cabbage) as a visual indicator of fish deterioration. LWT-Food Sci. Technol..

[118] Kannan S.K., Ambrose B., Sudalaimani S., Pandiaraj M., Giribabu K., Kathiresan M. (2020). A review on chemical and electrochemical methodologies for the sensing of biogenic amines. Anal. Methods.

[119] Arabi M., Ostovan A., Li J., Wang X., Zhang Z., Choo J., Chen L. (2021). Molecular imprinting: Green perspectives and strategies. Adv. Mater..

[120] Wang L., Pagett M., Zhang W. (2023). Molecularly imprinted polymer (MIP) based electrochemical sensors and their recent advances in health applications. Sens. Actuators Rep..

[121] Modesti M., Taglieri I., Bianchi A., Tonacci A., Sansone F., Bellincontro A., Venturi F., Sanmartin C. (2021). E-nose and olfactory assessment: Teamwork or a challenge to the last data? The case of virgin olive oil stability and shelf life. Appl. Sci..

[122] Sanislav T., Mois G.D., Zeadally S., Folea S., Radonić T.C., Al-Suhaimi E.A. (2025). Review on sensor-based electronic nose for food quality. Sensors.

[123] Leon-Medina J.X., Anaya M., Tibaduiza D.A. (2022). Yogurt classification using an electronic tongue system and machine learning techniques. Intell. Syst. Appl..

[124] Garcia-Hernandez C., Garcia-Cabezon C., Rodriguez-Mendez M.L., Martin-Pedrosa F. (2025). Electronic tongue technology applied to grapes and wines: A review. Chemosensors.

[125] Ahari H., Jafari A., Ozdal T., Moradi S., Bahari H.R., Wu Q., Khaneghah A.M. (2025). Recent innovations in metal-based nanoparticles for food packaging: A focus on safety and environmental impact. Appl. Food Res..

[126] Okur E.E., Eker F., Akdaşçi E., Bechelany M., Karav S. (2025). Comprehensive review of silver nanoparticles in food packaging applications. Int. J. Mol. Sci..

[127] Anh N.H., Doan M.Q., Dinh N.X., Huy T.Q., Tri D.Q., Loan L.T.N., Le A.T. (2022). Gold nanoparticle-based optical nanosensors for food and health safety monitoring: Recent advances and future perspectives. RSC Adv..

[128] Rastogi S., Kumari V., Sharma V., Ahmad F.J. (2022). Gold nanoparticle-based sensors in food safety applications. Food Anal. Methods.

[129] Aldewachi H., Chalati T., Woodroofe M.N., Bricklebank N., Sharrack B., Gardiner P. (2018). Gold nanoparticle-based colorimetric biosensors. Nanoscale.

[130] Yáñez-Sedeño P., Pingarrón J.M. (2005). Gold nanoparticle-based electrochemical biosensors. Anal. Bioanal. Chem..

[131] Yang M., Kostov Y., Bruck H.A., Rasooly A. (2009). Gold nanoparticle-based enhanced chemiluminescence immunosensor for detection of staphylococcal enterotoxin B in food. Int. J. Food Microbiol..

[132] Kumar N., Seth R., Kumar H. (2014). Colorimetric detection of melamine in milk by citrate-stabilized gold nanoparticles. Anal. Biochem..

[133] Zheng F., Wang P., Du Q., Chen Y., Liu N. (2019). Simultaneous and ultrasensitive detection of foodborne bacteria by gold nanoparticles-amplified microcantilever array biosensor. Front. Chem..

[134] Duan X., Li Z., Wang L., Lin H., Wang K. (2023). Engineered nanomaterials-based sensing systems for assessing the freshness of meat and aquatic products: A state-of-the-art review. Compr. Rev. Food Sci. Food Saf..

[135] El-Nour K.M.A., Salam E.T.A., Soliman H.M., Orabi A. (2017). Gold nanoparticles as a direct and rapid sensor for sensitive analytical detection of biogenic amines. Nanoscale Res. Lett..

[136] Danchuk A.I., Komova N.S., Mobarez S.N., Doronin S.Y., Burmistrova N.A., Markin A.V., Duerkop A. (2020). Optical sensors for determination of biogenic amines in food. Anal. Bioanal. Chem..

[137] El-Nour K.M.M.A., Eftaiha A., Al-Warthan A., Ammar R.A.A. (2010). Synthesis and applications of silver nanoparticles. Arab. J. Chem..

[138] Bouafia A., Laouini S.E., Ahmed A.S., Soldatov A.V., Algarni H., Feng Chong K., Ali G.A. (2021). The recent progress on silver nanoparticles: Synthesis and electronic applications. Nanomaterials.

[139] Alberti G., Zanoni C., Magnaghi L.R., Biesuz R. (2021). Gold and silver nanoparticle-based colorimetric sensors: New trends and applications. Chemosensors.

[140] Caro C., Castillo P.M., Klippstein R., Pozo D., Zaderenko A.P., Alarcon E.I., Griffith M., Udekwu K.I. (2010). Silver nanoparticles: Sensing and imaging applications. Silver Nanoparticles.

[141] Magdy G., Aboelkassim E., Elhaleem S.M.A., Belal F. (2024). A comprehensive review on silver nanoparticles: Synthesis approaches, characterization techniques, and recent pharmaceutical, environmental, and antimicrobial applications. Microchem. J..

[142] Ivanišević I., Milardović S., Kassal P. (2021). Recent Advances in (Bio)Chemical Sensors for Food Safety and Quality Based on Silver Nanomaterials. Food Technol. Biotechnol..

[143] Torre R., Costa-Rama E., Nouws H.P.A., Delerue-Matos C. (2020). Screen-printed electrode-based sensors for food spoilage control: Bacteria and biogenic amines detection. Biosensors.

[144] Appendini P., Hotchkiss J.H. (2002). Review of antimicrobial food packaging. Innov. Food Sci. Emerg. Technol..

[145] Bittar D.B., Catelani T.A., Nigoghossian K., Barud H.D.S., Ribeiro S.J.L., Pezza L., Pezza H.R. (2017). Optimized synthesis of silver nanoparticles by factorial design with application for the determination of melamine in milk. Anal. Lett..

[146] Omole R., Torimiro N., Alayande S., Ajenifuja E. (2018). Silver nanoparticles synthesized from *Bacillus subtilis* for detection of deterioration in the post-harvest spoilage of fruit. Sustain. Chem. Pharm..

[147] EFSA Panel on Food Contact Materials, Enzymes and Processing Aids (CEP) (2021). Guidance on Risk Assessment of Nanomaterials to Be Applied in the Food and Feed Chain. EFSA J..

[148] Şerban I., Enesca A. (2020). Metal oxides-based semiconductors for biosensors applications. Front. Chem..

[149] Ramadan K.M., Bendary E.S., Khalil H.B., Ali S.A., Ahmed A.R., Mahmoud M.A. (2025). Smart detection of food spoilage using microbial volatile compounds: Technologies, challenges, and future outlook. J. Agric. Food Chem..

[150] Ivanov A., Buruiana D.L., Trus C., Ghisman V., Vasile Antoniac I. (2025). Nanomaterials for sensory systems—A review. Biosensors.

[151] Zhang Y., Zhao Y., Jiang F., Lai R. (2025). Design of electronic nose based on MOS gas sensors and its application in juice identification. Sensors.

[152] Fletcher B., Mullane K., Platts P., Todd E., Power A., Roberts J., Chandra S. (2018). Advances in meat spoilage detection: A short focus on rapid methods and technologies. CyTA J. Food.

[153] Ajaykumar V.J., Mandal P.K., Biswas A.K., Mandal P.K. (2020). Modern concept and detection of spoilage in meat and meat products. Meat Quality Analysis.

[154] Veerapandi G., Lavanya N., Neri G., Sekar C. (2025). Non-enzymatic electrochemical sensors based on nanostructured metal oxides for food quality assessment: A review. Trends Food Sci. Technol..

[155] Speranza G. (2021). Carbon nanomaterials: Synthesis, functionalization and sensing applications. Nanomaterials.

[156] Campuzano S., Yáñez-Sedeño P., Pingarrón J.M. (2019). Carbon dots and graphene quantum dots in electrochemical biosensing. Nanomaterials.

[157] Pan M., Yin Z., Liu K., Du X., Liu H., Wang S. (2019). Carbon-based nanomaterials in sensors for food safety. Nanomaterials.

[158] Lei J., Ju H. (2012). Signal amplification using functional nanomaterials for biosensing. Chem. Soc. Rev..

[159] Vasilescu A., Hayat A., Gáspár S., Marty J.L. (2018). Advantages of carbon nanomaterials in electrochemical aptasensors for food analysis. Electroanalysis.

[160] Pathak A.K., Swargiary K., Kongsawang N., Jitpratak P., Ajchareeyasoontorn N., Udomkittivorakul J., Viphavakit C. (2023). Recent advances in sensing materials targeting clinical volatile organic compound (VOC) biomarkers: A review. Biosensors.

[161] Greco E., De Spirt A., Miani A., Piscitelli P., Trombin R., Barbieri P., Marin E. (2025). Nanomaterials in photocatalysis: An in-depth analysis of their role in enhancing indoor air quality. Appl. Sci..

[162] Iravani S., Varma R.S. (2020). Green synthesis, biomedical and biotechnological applications of carbon and graphene quantum dots. A review. Environ. Chem. Lett..

[163] Machín A., Márquez F. (2025). Next-generation chemical sensors: The convergence of nanomaterials, advanced characterization, and real-world applications. Chemosensors.

[164] Zhang H., Chan-Park M.B., Wang M. (2020). Functional polymers and polymer–dye composites for food sensing. Macromol. Rapid Commun..

[165] Idumah C.I., Hassan A., Ihuoma D.E. (2019). Recently emerging trends in polymer nanocomposites packaging materials. Polym.-Plast. Technol. Mater..

[166] Saini P., Patel P., Chatterjee H., Biswas S., Bhati S.S., Dutt D. (2025). Polymer-based composites for future packaging materials. Polymers and Composite Materials for Packaging: Smart Food Packaging and Solutions.

[167] Yam K.L., Takhistov P.T., Miltz J. (2005). Intelligent packaging: Concepts and applications. J. Food Sci..

[168] Subhashree S., Kumar P.S. (2022). New analytical strategies amplified with carbon-based nanomaterial for sensing food pollutants. Chemosphere.

[169] Raja V., Kour G., Katal D., Kaur S., Malik A.Q. (2026). Carbon-based nanocomposites for sensing and biosensing. Carbon-Based Nanocomposites for Sustainable Applications, Volume III: Biomedicine, Electronics, and Catalysis.

[170] Singh S., Gaikwad K.K., Lee Y.S. (2018). Anthocyanin—A natural dye for smart food packaging systems. Korean J. Packag. Sci. Technol..

[171] Ranvare A.R., Singh A., Khule G.D. (2026). Advanced packaging solutions for quality maintenance. Food Quality Assurance.

[172] Hu Z., Zhao R., Wang F., Ren L., Wang L., Jiang L. (2026). Stimuli-responsive hydrogels in food sector: Multi-component design, stimulus-response mechanisms, and broad applications. Gels.

[173] Lin X., Wang Z., Jia X., Chen R., Qin Y., Bian Y., Gao Z. (2023). Stimulus-responsive hydrogels: A potent tool for biosensing in food safety. Trends Food Sci. Technol..

[174] Zhai X., Zou X., Shi J., Huang X., Sun Z., Li Z., Huang X., Holmes M., Gong Y. (2017). Novel colorimetric films based on starch/polyvinyl alcohol incorporated with roselle anthocyanins for fish freshness monitoring. Food Hydrocoll..

[175] Gopalakrishnan K., Bayan A., Mishra P. (2025). Comprehensive review on integration of self-healing polymers with smart food traceability: Scope, application and challenges. Sustain. Food Technol..

[176] Piramuthu S., Zhou W. (2016). RFID and Sensor Network Automation in the Food Industry: Ensuring Quality and Safety through Supply Chain Visibility.

[177] Virumbrales C., Hernández-Ruiz R., Trigo-López M., Vallejos S., García J.M. (2024). Sensory polymers: Trends, challenges, and prospects ahead. Sensors.

[178] Turner A.P.F. (2013). Biosensors: Sense and sensibility. Chem. Soc. Rev..

[179] Sonowal K., Borthakur P.P., Pathak K. (2025). Advances in enzyme-based biosensors: Emerging trends and applications. Eng. Proc..

[180] Wijayanti S.D., Tsvik L., Haltrich D. (2023). Recent advances in electrochemical enzyme-based biosensors for food and beverage analysis. Foods.

[181] Dzyadevych S.V., Soldatkin A.P., El’skaya A.V., Martelet C., Jaffrezic-Renault N. (2006). Enzyme biosensors based on ion-selective field-effect transistors. Anal. Chim. Acta.

[182] Sharma N.K., Monika, Kaushal A., Thakur S., Thakur N., Sheetal, Bhalla T.C. (2021). Nanohybrid electrochemical enzyme sensor for xanthine determination in fish samples. 3 Biotech.

[183] Datta S., Christena L.R., Rajaram Y.R.S. (2013). Enzyme immobilization: An overview on techniques and support materials. 3 Biotech.

[184] Kumar H., Ahlawat Y.K., Sharma N., Dhalaria R., Sethi N., Guleria S., Kuča K. (2025). Applications of antibody-based sensors in the food sector. Applications of Biosensors in Healthcare.

[185] Duffy G.F., Moore E.J. (2017). Electrochemical immunosensors for food analysis: A review of recent developments. Anal. Lett..

[186] Prajapati V., Shrivastav P. (2023). Bioreceptors for antigen–antibody interactions. Biosensors Nanotechnology.

[187] Hasanzadeh M., Shadjou N. (2017). Advanced nanomaterials for use in electrochemical and optical immunoassays of carcinoembryonic antigen. A review. Microchim. Acta.

[188] Piletsky S.A., Turner A.P.F. (2002). Electrochemical sensors based on molecularly imprinted polymers. Electroanalysis.

[189] Ashraf S., Mustafa G., Pervaiz K., Altaf A.A., Hamayun M. (2026). Smart recognition of chemical contaminants in food: Experimental and computational perspectives on MIP-based sensors. Anal. Methods.

[190] Ahmed S., Ansari A., Li Z., Mazumdar H., Siddiqui M.A., Khan A., Ranjan P., Kaushik A., Vinu A., Kumar P. (2025). Point-of-care health diagnostics and food quality monitoring by molecularly imprinted polymers-based histamine sensors. Adv. Sens. Res..

[191] Fu Q.B., Sun X., Liu L., Zhao R.S., Wang X. (2025). Current progress of molecularly imprinted polymers for emerging pollutants analysis in food. J. Agric. Food Chem..

[192] Singh S., Sharma P., Thakur N. (2026). Towards safer food systems: Emerging biosensing strategies for food quality, contamination, and authenticity. Food Nutr. Health.

[193] Dey A. (2021). Semiconductor metal oxide gas sensors: A review. Mater. Sci. Eng. B.

[194] Joshi N., Pransu G., Conte-Junior C.A. (2023). Critical review and recent advances of 2D materials-based gas sensors for food spoilage detection. Crit. Rev. Food Sci. Nutr..

[195] Askim J.R., Mahmoudi M., Suslick K.S. (2021). Optical sensor arrays for chemical detection: The optoelectronic nose. Chem. Soc. Rev..

[196] Meléndez F., Sánchez R., Fernández J.A., Belacortu Y., Bermúdez F., Arroyo P., Lozano J. (2023). Design of a multisensory device for tomato volatile compound detection based on a mixed metal oxide–electrochemical sensor array and optical reader. Micromachines.

[197] Mia N. (2026). Volatile fingerprinting and analytical strategies for raw meat authentication in processed meat products: A comprehensive review. Food Wellness.

[198] Sawant S.S., Park H.-Y., Sim E.-Y., Kim H.-S., Choi H.-S. (2025). Microbial Fermentation in Food: Impact on Functional Properties and Nutritional Enhancement—A Review of Recent Developments. Fermentation.

[199] Zhang Y.M., Gao P., Zhang W.Y., Zhu H., Wang C., Xie N., Wang Y., Pang X. (2023). Free fatty acid hydrolyzed with lipases and their effects on enzyme-modified cheese flavor. Food Sci. Anim. Prod..

[200] Zhou W., Yang Q., Tao S., Cui J., Zhu J., Zhou S., Li R., Su J., Zhang N., Xu L. (2024). Utilization of Tea Polyphenols as Color Developers in Reversible Thermochromic Dyes for Thermosensitive Color Change and Enhanced Functionality of Polyester Fabrics. Molecules.

[201] Doğan V., Evliya M., Kahyaoglu L.N., Kılıç V. (2024). On-site colorimetric food spoilage monitoring with smartphone-embedded machine learning. Talanta.

[202] Jia X., Ma P., Tarwa K., Mao Y., Wang Q. (2023). Development of a novel colorimetric sensor array based on oxidized chitin nanocrystals and deep learning for monitoring beef freshness. Sens. Actuators B Chem..

[203] Ghorbanizamani F. (2025). A combinatorial approach to chicken meat spoilage detection using color-shifting silver nanoparticles, smartphone imaging, and artificial neural network (ANN). Food Chem..

[204] Gong W., Yao H.B., Chen T., Xu Y., Fang Y., Zhang H.Y., Li B.W., Hu J.N. (2023). Smartphone platform based on gelatin methacryloyl (GelMA) combined with deep learning models for real-time monitoring of food freshness. Talanta.

[205] Kuswandi B., Jayus, Restyana A., Abdullah A., Heng L.Y., Ahmad M. (2012). A novel colorimetric food package label for fish spoilage based on polyaniline film. Food Control.

[206] Prietto L., Mirapalhete T.C., Pinto V.Z., Hoffmann J.F., Vanier N.L., Lim L.T., Dias A.R.G. (2017). pH-sensitive films containing anthocyanins extracted from black bean seed coat and red cabbage. LWT.

[207] Pereira V.A., Arruda I.N.Q., Stefani R. (2015). Active chitosan/PVA films with anthocyanins from *Brassica oleracea* (red cabbage) as time–temperature indicators for intelligent food packaging. Food Hydrocoll..

[208] Gram L., Dalgaard P. (2002). Fish spoilage bacteria—Problems and solutions. Curr. Opin. Biotechnol..

[209] Chen H.-Z., Zhang M., Bhandari B., Guo Z. (2018). Applicability of a colorimetric indicator label for monitoring freshness of fresh-cut green bell pepper. Postharvest Biol. Technol..

[210] Feng L., Liu Y., Wang Y., Zhou H., Lu Z., Li T. (2024). Ultra-compact dual-channel integrated CO_2_ infrared gas sensor. Microsyst. Nanoeng..

[211] Ma M., Yang X., Ying X., Shi C., Jia Z., Jia B. (2023). Applications of Gas Sensing in Food Quality Detection: A Review. Foods.

[212] Bruce J., Ouellette C., Bosnick K. (2020). Zinc oxide sensing devices for meat spoilage detection. ECS Meet. Abstr..

[213] Albarakati A.J., Matter I.A. (2023). N-Type Metal Oxide Semiconductor: Materials and Their Environmental Applications. Biointerface Res. Appl. Chem..

[214] Ataei Kachouei M., Kaushik A., Ali M.A. (2023). Internet of things-enabled food and plant sensors to empower sustainability. Adv. Intell. Syst..

[215] Melikoglu M. (2025). The Electronic Nose: A Critical Global Review of Advances in Analytical Methods and Real-World Applications. Microchem. J..

[216] Ha D.T.T., Dinh Van N., Nguyen Duc H. (2026). Electronic Nose in Gas Sensing: From Sensors to Artificial Intelligence: A Review. Sens. Rev..

[217] Vanaraj R., Bincy I.P., Mayakrishnan G., Kim I.-S., Kim S. (2025). A Systematic Review of the Applications of Electronic Nose and Electronic Tongue in Food Quality Assessment and Safety. Chemosensors.

[218] Gancarz M., Malaga-Toboła U., Oniszczuk A., Tabor S., Oniszczuk T., Gawrysiak-Witulska M., Rusinek R. (2021). Detection and measurement of aroma compounds with the electronic nose during bread production. Food Bioprod. Process..

[219] Gondaliya S.M., Sheth J., Shah S. (2024). Exploring the culinary and health significance of fermented foods: A comprehensive review. World J. Adv. Res. Rev..

[220] Abbaspour N. (2024). Fermentation’s pivotal role in shaping the future of plant-based foods: An integrative review of fermentation processes and their impact on sensory and health benefits. Appl. Food Res..

[221] Seesaard T., Wongchoosuk C. (2022). Recent progress in electronic noses for fermented foods and beverages applications. Fermentation.

[222] Deng L., Zhao X., Chen L. (2025). Microbial volatile organic compounds for food quality and safety: Metabolic pathways, sampling and detection, and machine learning-driven insights. Trends Food Sci. Technol..

[223] Mefleh M., Darwish A.M.G., Mudgil P., Maqsood S., Boukid F. (2022). Traditional fermented dairy products in southern Mediterranean countries: From tradition to innovation. Fermentation.

[224] Marco M.L., Heeney D., Binda S., Cifelli C.J., Cotter P.D., Foligné B., Gänzle M., Kort R., Pasin G., Pihlanto A. (2017). Health benefits of fermented foods: Microbiota and beyond. Curr. Opin. Biotechnol..

[225] Şanlier N., Gökcen B.B., Sezgin A.C. (2019). Health benefits of fermented foods. Crit. Rev. Food Sci. Nutr..

[226] Sessou P., Keisam S., Gagara M., Komagbe G., Farougou S., Mahillon J., Kumaraswamy J. (2023). Comparative analyses of bacterial communities present in spontaneously fermented milk products of Northeast India and West Africa. Front. Microbiol..

[227] Fernández M., Hudson J.A., Korpela R., de los Reyes-Gavilán C.G. (2015). Impact on human health of microorganisms present in fermented dairy products: An overview. Biomed. Res. Int..

[228] Martin N.H., Torres-Frenzel P., Wiedmann M. (2021). Invited review: Controlling dairy product spoilage to reduce food loss and waste. J. Dairy Sci..

[229] Shaker R., Jumah R., Abu-Jdayil B. (2000). Rheological Properties of Plain Yogurt during Coagulation Process: Impact of Fat Content and Preheat Treatment of Milk. J. Food Eng..

[230] Bansal V., Veena N. (2024). Understanding the role of pH in cheese manufacturing: General aspects of cheese quality and safety. J. Food Sci. Technol..

[231] Meenakshi P.L., Chaturvedi S., Annamalai M. (2025). Non-destructive testing techniques (NDTTs) for microbial contamination in cheese: A review. Trends Food Sci. Technol..

[232] Hristovski K.D., Burge S.R., Boscovic D., Burge R.G., Babanovska-Milenkovska F. (2022). Real-time monitoring of kefir-facilitated milk fermentation using microbial potentiometric sensors. J. Environ. Chem. Eng..

[233] Zeng H., Han H., Huang Y., Wang B. (2023). Rapid Prediction of the Aroma Type of Plain Yogurts via Electronic Nose Combined with Machine Learning Approaches. Food Biosci..

[234] Tonacci A., Scafile A., Billeci L., Sansone F. (2022). Electronic Nose and Tongue for Assessing Human Microbiota. Chemosensors.

[235] Saetae D. (2025). Machine learning-based prediction of microbial growth and acidification in yogurt fermentation at industrial temperatures. LWT.

[236] Ray A., Sinha C., Sharma A.K., Khamrui K., Hussain S.A., Dabas J.K., Mohanty T.K. (2026). AI-driven real-time monitoring and predictive control system for yoghurt fermentation. Food Control.

[237] Huang Q., Ye H., Yang Y., Zhu C., Tang J. (2025). Physicochemical Properties, Antioxidant Activities, and Aromatic Profile of Yogurt Co-Fermented by Weissella cibaria G232 with Traditional Starters. Foods.

[238] Campos-Góngora E., González-Martínez M.T., López-Hernández A.A., Arredondo-Mendoza G.I., Ortega-Villarreal A.S., González-Martínez B.E. (2023). Histamine and tyramine in Chihuahua cheeses during shelf life: Association with the presence of *tdc* and *hdc* genes. Molecules.

[239] Natrella G., Vacca M., Minervini F., Faccia M., De Angelis M. (2024). A comprehensive review on the biogenic amines in cheeses: Their origin, chemical characteristics, hazard and reduction strategies. Foods.

[240] Verma N., Hooda V., Gahlaut A., Gothwal A., Hooda V. (2019). Enzymatic Biosensors for the Quantification of Biogenic Amines: A Literature Update. Crit. Rev. Biotechnol..

[241] Li X., Wu Z., Cao H., Ye T., Hao L., Yu J., Yuan M., Xu F. (2025). A Molecularly Imprinted Fluorescence Sensor for the Simultaneous and Rapid Detection of Histamine and Tyramine in Cheese. Foods.

[242] Givanoudi S., Heyndrickx M., Depuydt T., Khorshid M., Robbens J., Wagner P. (2023). A Review on Bio- and Chemosensors for the Detection of Biogenic Amines in Food Safety Applications: The Status in 2022. Sensors.

[243] Adedeji A.A., Ekramirad N., Rady A., Hamidisepehr A., Donohue K.D., Villanueva R.T., Parrish C.A., Li M. (2020). Non-destructive technologies for detecting insect infestation in fruits and vegetables under postharvest conditions: A critical review. Foods.

[244] Han H., Li M., Peng Y., Zhang Z., Yue X., Zheng Y. (2021). Microbial diversity and non-volatile metabolites profile of low-temperature sausage stored at room temperature. Front. Microbiol..

[245] Lv Y., Yin X., Wang Y., Chen Q., Kong B. (2021). Prediction of specific spoilage organisms in Harbin red sausage stored at room temperature by multivariate statistical analysis. Food Control.

[246] Chen Q., Hu Y., Wen R., Wang Y., Qin L., Kong B. (2021). Characterisation of the Flavour Profile of Dry Fermented Sausages with Different NaCl Substitutes Using HS-SPME-GC-MS Combined with Electronic Nose and Electronic Tongue. Meat Sci..

[247] de Lima Alves L., Donadel J.Z., Athayde D.R., da Silva M.S., Klein B., Fagundes M.B., de Menezes C.R., Barin J.S., Campagnol P.C.B., Wagner R. (2020). Effect of ultrasound on proteolysis and formation of volatile compounds in dry fermented sausages. Ultrason. Sonochem..

[248] Hu Y., Tian Y., Zhu J., Wen R., Chen Q., Kong B. (2022). Technological characterization and flavor-producing potential of lactic acid bacteria isolated from traditional dry fermented sausages in Northeast China. Food Microbiol..

[249] Gao F., Zhang K., Wang D., Xia L., Gu Y., Tian J., Jin Y. (2024). Effect of *Lactobacillus helveticus* IMAUJBH1 on fat and volatile flavor substances in fermented mutton sausages. Food Chem. X.

[250] Alam A.M.M.N., Mia N., Monti J.A., Hashem M.A., Ali M.S. (2024). Enhancing the qualitative attributes of meat through processing and preservation techniques: A review. Meat Res..

[251] Liang J., Li D., Shi R., Wang J., Ma Y., Xiong K. (2019). Effects of different co-cultures on amino acid availability, biogenic amine concentrations and protein metabolism of fermented sufu and their relationships. LWT.

[252] Shao L., Sun Y., Zou B., Zhao Y., Li X., Dai R. (2023). Sublethally injured microorganisms in food processing and preservation: Quantification, formation, detection, resuscitation and adaptation. Food Res. Int..

[253] Ma Y., Gao Y., Xu Y., Zhou H., Zhou K., Li C., Xu B. (2023). Microbiota dynamics and volatile metabolite generation during sausage fermentation. Food Chem..

[254] Zamfir L.-G., Răut I., Constantin M., Corneli N.O., Firincă C., Jecu M.-L., Epure P., Nistor C.L., Doni M., Gurban A.-M. (2025). Assessment of biogenic amines produced by microorganisms as food spoilage indicators by sensitive detection using portable opto-electrochemical tools based on biosensors. Food Control.

[255] Ailer Š., Jakabová S., Benešová L., Ivanova-Petropulos V. (2022). Wine faults: Reductive aromas, oxidation and atypical aging. Molecules.

[256] Müller N., Rauhut D., Tarasov A. (2022). Sulfane sulfur compounds and reductive off-odors in wine. Fermentation.

[257] Habschied K., Krstanović V., Mastanjević K. (2022). Beer Quality Evaluation—A Sensory Aspect. Beverages.

[258] Rodríguez-Méndez M.L., de Saja J.A., González-Antón R., García-Hernández C., Medina-Plaza C., García-Cabezón C., Martín-Pedrosa F. (2016). Electronic noses and tongues in wine industry. Front. Bioeng. Biotechnol..

[259] Liu H., Li Q., Yan B., Zhang L., Gu Y. (2019). Bionic electronic nose based on MOS sensors and machine learning. Sensors.

[260] Freddi S., Bollani M., Sangaletti L. (2026). Wine discrimination through an electronic nose based on SWCNTs functionalized with Zn phthalocyanine. Sens. Actuators B Chem..

[261] Liu L., Na N., Yu J., Zhao W., Wang Z., Zhu Y., Hu C. (2023). Sniffing like a wine taster: MOSS strategy enhances electronic nose capability. Adv. Sci..

[262] Alfieri G., Modesti M., Riggi R., Bellincontro A. (2024). Recent advances in e-nose technologies in wine industry. Sensors.

[263] Muñoz-Castells R., Modesti M., Moreno-García J., Rodríguez-Moreno M., Catini A., Capuano R., Di Natale C., Bellincontro A., Moreno J. (2025). Differentiation of rosé sparkling wines using e-nose and GC-FID. J. Sci. Food Agric..

[264] Santos J.P., Sayago I., Sanjurjo J.L., Perez-Coello M.S., Díaz-Maroto M.C. (2022). Rapid analysis of corky off-flavors using a portable electronic nose. Sensors.

[265] Meléndez F., Arroyo P., Gómez-Suárez J., Palomeque-Mangut S., Suárez J.I., Lozano J. (2022). Portable electronic nose for TCA discrimination. Sensors.

[266] Franceschi D., Cavalet E., Boatto V., Conte G., Bravi M. (2016). Artificial diagnosis of *Brettanomyces* contamination. Chem. Eng. Trans..

[267] Lozano J., Santos J.P., Suárez J.I., Cabellos M., Arroyo T., Horrillo C. (2015). Automatic sensor system for the continuous analysis of the evolution of wine. Am. J. Enol. Vitic..

[268] Martínez-García R., Moreno J., Bellincontro A., Centioni L., Puig-Pujol A., Peinado R.A., Mauricio J.C., García-Martínez T. (2021). Using an electronic nose and volatilome analysis to differentiate sparkling wines obtained under different conditions. Food Chem..

[269] Ma Y., Yu K., Chen X., Wu H., Xiao X., Xie L., Wei Z., Xiong R., Zhou X. (2023). Effects of plant-derived polyphenols on wine aroma. Molecules.

[270] Gamboa J.C.R., da Silva A.J., de Andrade Lima L.L., Ferreira T.A.E. (2019). Rapid detection of wine spoilage using electronic nose. LWT.

[271] Gamboa J.C.R., da Silva A.J., Araujo I.C., Albarracin E.E.S., Duran A.C.M. (2021). Validation of a rapid detection approach to enhance electronic nose performance using deep learning and SVM. Sens. Actuators B Chem..

[272] Gonzalez Viejo C., Fuentes S. (2022). Digital assessment and classification of wine faults using a low-cost electronic nose, near-infrared spectroscopy and machine learning modelling. Sensors.

[273] Aleixandre M., Cabellos J.M., Arroyo T., Horrillo M.C. (2018). Quantification of wine mixtures using electronic nose and sensory panel. Front. Bioeng. Biotechnol..

[274] Georgiadou E.C., Mina M., Neoptolemou V., Koundouras S., D’Onofrio C., Bellincontro A., Mencarelli F., Fotopoulos V., Manganaris G.A. (2023). Effect of leaf removal on physiological and volatile profiles of grape cultivar Xynisteri. J. Sci. Food Agric..

[275] Bianchi A., Santini G., Piombino P., Pittari E., Sanmartin C., Moio L., Modesti M., Bellincontro A., Mencarelli F. (2023). Nitrogen maceration as an alternative to carbonic maceration. Food Chem..

[276] Modesti M., Alfieri G., Chieffo C., Mencarelli F., Vannini A., Catalani A., Chilosi G., Bellincontro A. (2023). Early detection of noble rot in wine grapes. J. Sci. Food Agric..

[277] Baietto M., Wilson A.D. (2015). Electronic nose applications for fruit quality. Sensors.

[278] Gardner D.M., Duncan S.E., Zoecklein B.W. (2017). Aroma characterization of Petit Manseng wines. Am. J. Enol. Vitic..

[279] Han F., Zhang D., Aheto J.H., Feng F., Duan T. (2020). Integration of electronic nose and tongue for red wine identification. Food Sci. Nutr..

[280] Paup V.D., Cook-Barton T., Diako C., Edwards C.G., Ross C.F. (2021). Detection of red wine faults using electronic tongue. Beverages.

[281] Potter R.I., Lee J., Ross C.F. (2025). Early oxidation detection in white wine by electronic tongue. Food Sci. Nutr..

[282] Marques C., Dinis L.-T., Modesti M., Bellincontro A., Correia E., Vilela A. (2024). Exploring the influence of terroir on Douro white and red wines characteristics: A study of human perception and electronic analysis. Eur. Food Res. Technol..

[283] Diako C., Vixie B., Weller K.M., Dycus D.A., Ross C.F. (2017). Determination of 4-ethylcatechol in Merlot wine using sensory and electronic tongue. Int. J. Food Sci. Technol..

[284] Prieto N., Gay M., Vidal S., Aagaard O., de Saja J.A., Rodríguez-Méndez M.L. (2011). Analysis of closure type influence on red wine organoleptic characteristics using an electronic panel. Food Chem..

[285] Rodríguez-Méndez M.L., Arrieta A.A., Parra V., Bernal A., Vegas A., Villanueva S., Gutiérrez-Osuna R., AntoniodeSaja J., de Saja J. (2004). Fusion of three sensory modalities for multimodal characterization of red wines. IEEE Sens. J..

[286] Oldham R.C., Held M.A. (2023). Methods for detection and identification of beer-spoilage microbes. Front. Microbiol..

[287] Piedra F.L., Villavicencio Yanos C.M., Mieles Mieles J., Cantos Macias M., Anchundia Loor A.M., Zambrano Vélez M.I., Intriago Flor F.G., Giler Intriago S.N., Pérez Vega E., Litardo Velásquez R.M. (2025). Quantification of lactic acid in wines using an amperometric biosensor. Food Control.

[288] Almeida C.M.R., Barroso M.F., Moreira M.M., Magalhães J.M.C.S., Durães L. (2025). Direct electrochemical detection of tyramine in beer samples. Sensors.

[289] Majer-Baranyi K., Székács A., Adányi N. (2023). Application of electrochemical biosensors for determination of food spoilage. Biosensors.

[290] Sanaeifar A., ZakiDizaji H., Jafari A., de la Guardia M. (2017). Early detection of contamination and defects in foodstuffs by electronic nose: A review. TrAC Trends Anal. Chem..

[291] Gonzalez Viejo C., Fuentes S., Hernández-Brenes C. (2021). Smart Detection of Faults in Beers Using Near-Infrared Spectroscopy, a Low-Cost Electronic Nose and Artificial Intelligence. Fermentation.

[292] Lima A.C., Aceña L., Mestres M., Boqué R. (2024). A rapid method to predict beer shelf life using an MS-based e-nose. Beverages.

[293] Nimsuk N. (2019). Improvement of beer classification using electronic nose technology. J. Food Meas. Charact..

[294] Zouharová A., Bartáková K., Bursová S., Necidová L., Harustiakova D., Klimešová M., Vorlová L. (2023). Meat and fish packaging and its impact on the shelf life—A review. Acta Vet. Brno.

[295] Ellis D.I., Broadhurst D., Kell D.B., Rowland J.J., Goodacre R. (2002). Rapid and quantitative detection of the microbial spoilage of meat by Fourier transform infrared spectroscopy and machine learning. Appl. Environ. Microbiol..

[296] Dashmaa D., Xi L., Van Ba H., Dawoon J., Hwang I. (2026). Flavoromics of meats as a function of postmortem proteolysis. Food Sci. Anim. Resour..

[297] Pellissery A.J., Vinayamohan P., Amalaradjou M., Venkitanarayanan K. (2020). Spoilage bacteria and meat quality. Encyclopedia of Meat Sciences.

[298] Casaburi A., Piombino P., Nychas G.J.E., Villani F., Ercolini D. (2015). Bacterial populations and the volatilome associated to meat spoilage. Food Microbiol..

[299] Gram L., Ravn L., Rasch M., Bruhn J.B., Christensen A.B., Givskov M. (2002). Food spoilage—Interactions between food spoilage bacteria. Int. J. Food Microbiol..

[300] Ruiz-Capillas C., Jiménez-Colmenero F. (2004). Biogenic amines in meat and meat products. Crit. Rev. Food Sci. Nutr..

[301] Wang X., Zheng Z. (2025). Mechanistic insights into fish spoilage and integrated preservation technologies. Appl. Sci..

[302] Jaffrès E., Lalanne V., Macé S., Cornet J., Cardinal M., Sérot T., Dousset X., Joffraud J.J. (2011). Sensory characteristics of spoilage and volatile compounds associated with bacteria isolated from cooked and peeled tropical shrimp. Food Microbiol..

[303] Huss H.H. (1995). Quality and Quality Changes in Fresh Fish.

[304] Wang C., Yin L., Zhang L., Xiang D., Gao R. (2010). Metal oxide gas sensors: Sensitivity and influencing factors. Sensors.

[305] Mendes R.G., Wróbel P.S., Bachmatiuk A., Sun J., Gemming T., Liu Z., Rümmeli M.H. (2018). Carbon nanostructures as a multi-functional platform for sensing applications. Chemosensors.

[306] Narayana G.P., Singh D.K., Chudasama M.H., Deotale S., Reddy N.B.P., Perumal T. (2025). Intelligent packaging of functional foods: A comprehensive review of its advances, recyclability, and regulatory frameworks. Bioresour. Technol. Rep..

[307] Zhao L., Liu Y., Zhao L., Wang Y. (2022). Anthocyanin-based pH-sensitive smart packaging films for monitoring food freshness. J. Agric. Food Res..

[308] Ermakova E.V., Bessmertnykh-Lemeune A. (2024). The optical sensing of volatile organic compounds using porphyrins. Chemosensors.

[309] Cicero A., Cammilleri G., Galluzzo F.G., Calabrese I., Pulvirenti A., Giangrosso G., Cicero N., Cumbo V., Vella A., MaCaluso A. (2020). Histamine in fish products randomly collected in Southern Italy: A 6-year study. J. Food Prot..

[310] Ampuero S., Bosset J.O. (2003). The electronic nose applied to dairy products: A review. Sens. Actuators B Chem..

[311] Buratti S., Grassia S., Ubaldi P.G., Pianezzola A., Benedetti S. (2025). E-nose-based control charts for fish freshness evaluation. Food Res. Int..

[312] Realini C.E., Marcos B. (2014). Active and intelligent packaging systems for a modern society. Meat Sci..

[313] Zhai X., Xue Y., Sun Y., Ma X., Ban W., Marappan G., Tahir H.E., Huang X., Wu K., Chen Z. (2025). Colorimetric food freshness indicators for intelligent packaging: Progress, shortcomings, and promising solutions. Foods.

[314] Lukács M., Tóth F., Horváth R., Solymos G., Alpár B., Varga P., Kertész I., Gillay Z., Baranyai L., Felföldi J. (2025). Advanced digital solutions for food traceability: Enhancing origin, quality, and safety through NIRS, RFID, blockchain, and IoT. J. Sens. Actuator Netw..

[315] Davidescu M.A., Pânzaru C., Mădescu B.M., Poroșnicu I., Simeanu C., Usturoi A., Matei M., Doliș M.G. (2025). Advances and challenges in smart packaging technologies for the food industry: Trends, applications, and sustainability considerations. Foods.

[316] Pindi M. (2025). Revolutionizing cold chain logistics: Leveraging IoT and AI for enhanced food safety and waste reduction. World J. Adv. Res. Rev..

[317] Saltveit M.E., Yahia E.M. (2019). Respiratory metabolism. Postharvest Physiology and Biochemistry of Fruits and Vegetables.

[318] Umeohia U., Olapade A.A. (2024). Physiological processes affecting postharvest quality of fresh fruits and vegetables. Asian Food Sci. J..

[319] Barth M., Hankinson T.R., Zhuang H., Breidt F., Paliyath G., Murr D.P., Handa A.K., Lurie S. (2010). Microbiological spoilage of fruits and vegetables. Postharvest Biology and Technology of Fruits, Vegetables, and Flowers.

[320] Yuan Q., Jiang Y., Yang Q., Li W., Gan G., Cai L., Li W., Qin C., Yu C., Wang Y. (2024). Mechanisms and control measures of low temperature storage-induced chilling injury to solanaceous vegetables and fruits. Front. Plant Sci..

[321] Zakaria L. (2026). An overview of major Penicillium species associated with plant diseases. J. Fungi.

[322] Grierson D., Seymour G.B., Poole M., Giovannoni J.J., Tucker G.A. (2013). The molecular biology and biochemistry of fruit ripening. The Molecular Biology and Biochemistry of Fruit Ripening.

[323] Tipu M.M.H., Sherif S.M. (2024). Ethylene and its crosstalk with hormonal pathways in fruit ripening: Mechanisms, modulation, and commercial exploitation. Front. Plant Sci..

[324] Brizzolara S., Manganaris G.A., Fotopoulos V., Watkins C.B., Tonutti P. (2020). Primary metabolism in fresh fruits during storage. Front. Plant Sci..

[325] Porat R., Fallik E., Brückner B., Wyllie S.G. (2008). Production of off-flavours in fruit and vegetables under fermentative conditions. Fruit and Vegetable Flavour: Recent Advances and Future Prospects.

[326] Zhang J., Zhang J., Zhang L., Xue Y., Zhang K. (2025). Mechanistic insights into vegetable color stability: Discoloration pathways and emerging protective strategies. Foods.

[327] Gidado M.J., Gunny A.A.N., Ali A., Korsten L., Devi M. (2026). Environmental and external determinants of postharvest storage: Impacts, challenges, and sustainable mitigation strategies. Food Chem. Adv..

[328] Sabu A., Awasthi K., Pandey K., Pandey H. (2026). Advances in food spoilage detection through gas sensing: From material insights to prospects for commercialisation. J. Agric. Food Res..

[329] Lamberty A., Kreyenschmidt J. (2022). Ambient parameter monitoring in fresh fruit and vegetable supply chains using Internet of Things-enabled sensor and communication technology. Foods.

[330] Balakrishnan P., Leema A., Leema A., Jothiaruna N., Bhaiyya M. (2025). Artificial intelligence for food safety: From predictive models to real-world safeguards. Trends Food Sci. Technol..

[331] de Araújo T.P., de Moraes M.M., Afonso C., Santos C., Rodrigues S.S.P. (2022). Food processing: Comparison of different food classification systems. Nutrients.

[332] Gopal N., Hill C., Ross P.R., Beresford T.P., Fenelon M.A., Cotter P.D. (2015). The prevalence and control of *Bacillus* and related spore-forming bacteria in the dairy industry. Front. Microbiol..

[333] Rahman M., Islam R., Hasan S., Zzaman W., Rana M.R., Ahmed S., Roy M., Sayem A., Matin A., Raposo A. (2022). A comprehensive review on bio-preservation of bread: An approach to adopt wholesome strategies. Foods.

[334] Deligeorgakis C., Magro C., Skendi A., Gebrehiwot H.H., Valdramidis V., Papageorgiou M. (2023). Fungal and toxin contaminants in cereal grains and flours: Systematic review and meta-analysis. Foods.

[335] Church I.J., Parsons A.L. (1995). Modified atmosphere packaging technology: A review. J. Sci. Food Agric..

[336] Zamora R., Hidalgo F.J. (2011). The Maillard reaction and lipid oxidation. Lipid Technol..

[337] Sai-Ut S., Indriani S., Srisakultiew N., Kingwascharapong P., Suriyarak S., Issara U., Phongthai S., Rawdkuen S., Pongsetkul J. (2025). The role of CO_2_ levels in high-oxygen modified atmosphere packaging on microbial communities of chilled goat meat during storage and their relationship with quality attributes. Foods.

[338] Robertson G.L. (2012). Food Packaging: Principles and Practice.

[339] Fardet A., Rock E. (2020). Ultra-processed foods and food system sustainability: What are the links?. Sustainability.

[340] Erickson M.C., Doyle M.P. (2017). The challenges of eliminating or substituting antimicrobial preservatives in foods. Annu. Rev. Food Sci. Technol..

[341] Biji K.B., Ravishankar C.N., Mohan C.O., Gopal T.K.S. (2015). Smart packaging systems for food applications: A review. J. Food Sci. Technol..

[342] Panou A., Lazaridis D.G., Karabagias I.K. (2025). Application of smart packaging on the preservation of different types of perishable fruits. Foods.

[343] Azeredo H.M.C., Correa D.S. (2021). Smart choices: Mechanisms of intelligent food packaging. Curr. Res. Food Sci..

[344] Verhoef L.P.B., Bouma K., Biesterbos J.W.H., Sijm D.T.H.M. (2025). Time-temperature indicators on food products. Food Risk Assess. Eur..

[345] Siciliano S., Lopresto C.G., Carnì D.L., Lamonaca F. (2025). Optical gas sensors in smart food bio-packaging: Innovation for monitoring product freshness and safety. Meas. Food.

[346] Ramezani G., Assadpour E., Zhang W., Jafari S.M. (2025). Carbon nanomaterial-based sensors for smart packaging of food products. Ind. Crops Prod..

[347] Chen Z., Zhang G., Zhang F. (2026). Multimodal AI for real-time food safety and quality: From sensors to foundation models, edge deployment, and regulation. Food Sci. Nutr..

[348] Balta I., Lemon J., Popescu C.A., McCleery D., Iancu T., Pet I., Dumitrescu G., Petculescu Ciochina L., Marcu D., Morariu F. (2026). Artificial intelligence in next-generation smart packaging for pH and spoilage monitoring in poultry products. Appl. Food Res..

[349] Mostaccio A., Bianco G.M., Marrocco G., Occhiuzzi C. (2023). RFID technology for Food Industry 4.0: A review of solutions and applications. IEEE J. Radio Freq. Identif..

[350] Djafar M.J., Elmatsani H.M., Munarso S.J., Firdaus J., Trihadi S.E.Y., Purwanta W., Sadikin B.S., Sahlan S., Nasruddin N., Lanjar L. (2026). Smart Packaging 4.0: A bibliometric analysis of sensor integration, food safety, and sustainability in food packaging systems. Sci. World J..

[351] Rahman M., Sobhan A., Sadak O. (2026). Emerging trends in intelligent packaging for tackling food waste in the modern food supply chain. Trends Food Sci. Technol..

[352] Hossain A. (2023). Do date codes cause food waste? Smart packaging might tackle the problem. J. Food Bioact..

[353] Akbari N., Razavi S.H., Esmaeili N., Amini Valashani A., Barzegar Marvasti F., Salehian R. (2026). Recent developments in bio-based smart food packaging: Incorporating innovation and sensitive technologies for improved production. Nutr. Food Sci..

[354] Dakhia Z., Russo M., Merenda M. (2025). AI-enabled IoT for food computing: Challenges, opportunities, and future directions. Sensors.

[355] Falasconi M., Pardo M., Sberveglieri G. (2020). Electronic nose for food quality control. Encyclopedia of Sensors.

[356] Silva H., Gomes R., Sousa C. (2025). Classifying Forest Supply Chain Traceability Data with an Ontological Approach. Procedia Comput. Sci..

[357] Cosme F., Rocha T., Marques C., Barroso J., Vilela A. (2025). Innovative approaches in sensory food science: From digital tools to virtual reality. Appl. Sci..

[358] Alali A., Al-Naji A., Chahl J. (2023). A review of machine learning methods for the detection of food spoilage using electronic nose systems. Sensors.

[359] Kummer A.M., Burg T.P., Hierlemann A. (2006). Transient Signal Analysis Using Complementary Metal Oxide Semiconductor Capacitive Chemical Microsensors. Anal. Chem..

[360] Cai M., Xu S., Zhou X., Lu H. (2024). Electronic Nose Humidity Compensation System Based on Rapid Detection. Sensors.

[361] Lozano J., Arroyo P., Suárez J.I. (2021). Portable electronic nose based on electrochemical sensors for food quality assessment. Sensors.

[362] Arroyo P., Meléndez F., Suárez J.I., Herrero J.L., Rodríguez S., Lozano J. (2020). Electronic nose IoT node for waste and food quality monitoring. Sensors.

[363] Yoo M. (2026). Low-Cost Gas Sensing and Machine Learning for Intelligent Refrigeration in the Built Environment. Buildings.

[364] Yan C. (2025). A review on spectral data preprocessing techniques for machine learning and quantitative analysis. iScience.

[365] Kurz W., Wang K., Bektas F., Zhu C., Kariper E., Kurz M., Jakobi M., Baranes D., Koch A.W. (2025). Dimensionality reduction in hyperspectral imaging using standard deviation-based band selection for efficient classification. Sci. Rep..

[366] Yuan Y., Ji Z., Fan Y., Xu Q., Shi C., Lyu J., Ertbjerg P. (2025). Deep learning-assisted fluorescence spectroscopy for food quality and safety analysis. Trends Food Sci. Technol..

[367] Southey N.L., Zhu R., Holscher H.D. (2026). Machine learning and artificial intelligence in nutrition research: Analytical methods, applications, and key considerations. J. Nutr..

[368] Bellincontro A., Modesti M., De Santis D., Mencarelli F. (2021). A portable and non-destructive device for real-time monitoring of post-harvest quality. Sensors.

[369] Melucci D., Zappi A., Locatelli M. (2022). E-nose and e-tongue for dairy products: Trends in data fusion. Food Control.

[370] Melucci D., Locatelli M., Zappi A., Casolari S. (2021). Potentiometric e-tongue for biogenic amines in cheese. Sensors.

[371] Liu Y., Zhang X., Wang L. (2025). Attention-based CNN for ammonia detection in seafood. J. Food Eng..

[372] Fang H., Sun Y., Kim T.H., Xu J., Deng Q. (2026). Implementing ResNet for Early Detection and Classification of Fish Skin Diseases. Indian J. Anim. Res..

[373] Sherin Z., Bashir O., Mondal I.H., Pawase P.A. (2026). Advances in Hyperspectral Imaging for Spoilage Detection in Meat and Poultry: A Non-Invasive Approach. Trends Food Sci. Technol..

[374] Zhao X., Ning W., Chen R., Wang H., Zhang G., Bi J., Hou H. (2025). Rapid non-destructive detection of pork freshness using visible-near-infrared spectroscopy based on convolutional neural network hybrid models. J. Food Comp. Anal..

[375] Arroyo P., Lozano J., Suárez J.I. (2023). Framework for food quality monitoring using IoT and machine learning. J. Food Eng..

[376] Masuduzzaman M., Hassini E., Rahaman M.F., Al Mashalah H. (2026). FreshTrack: An Innovative IoT-Sensor-Driven Food Freshness Estimation Framework Integrating Blockchain. Sci. Rep..

[377] Modesti M., Bellincontro A., Modesti G. (2022). Evaluation of freshness in horticultural products using NIR and e-nose. Postharvest Biol. Technol..

[378] Gomes R., Ribeiro J.P., Silva R.G., Soares R. (2026). AI-Enabled Flexible Design of Resilient Forest-to-Bioenergy Supply Chains Under Wildfire Disruption Risk. Sustainability.

[379] Naik A., Lee H., Herrington J., Barandun G., Flock G., Güder F., Gonzalez-Macia L. (2024). Smart packaging with disposable NFC-enabled wireless gas sensors for monitoring food spoilage. ACS Sens..

[380] Istif E., Mirzajani H., Dağ Ç., Mirlou F., Ozuaciksoz E., Cakır C., Koydemir H., Yilgor I., Yilgor E., Beker L. (2023). Miniaturized wireless sensor enables real-time monitoring of food spoilage. Nat. Food.

[381] Zhang R., Wang M., Zhu T., Wan Z., Chen X., Xiao X. (2024). Wireless Charging Flexible In-Situ Optical Sensing for Food Monitoring. Chem. Eng. J..

[382] Luo D., Nikitina M., Xiao X. (2024). Flexible sensors for food monitoring. Part II: Applications. Food Syst..

[383] Hashemian H., Ghaedi M., Dashtian K., Mosleh S., Hajati S., Razmjoue D., Khan S. (2023). Cellulose acetate/MOF film-based colorimetric ammonia sensor for non-destructive remote monitoring of meat product spoilage. Int. J. Biol. Macromol..

[384] Hashemian H., Ghaedi M., Dashtian K., Khan S., Mosleh S., Hajati S., Razmjoue D. (2024). Highly sensitive fluorometric ammonia detection utilizing *Solenostemon scutellarioides* extracts in MOF-tragacanth gum hydrogel for meat spoilage monitoring. Sens. Actuators B Chem..

[385] Khan S., Prasad A., Javed M., MacLachlan R., Filipe C., Didar T. (2025). Food-activated microneedle sensor for real-time, colorimetric spoilage monitoring of pre-packaged food. Adv. Sci..

[386] Khan S., Monteiro J., Prasad A., Filipe C., Li Y., Didar T. (2023). Material breakthroughs in smart food monitoring: Intelligent packaging and on-site testing technologies for spoilage and contamination detection. Adv. Mater..

[387] Poghossian A., Geissler H., Schöning M. (2019). Rapid methods and sensors for milk quality monitoring and spoilage detection. Biosens. Bioelectron..

[388] Jafarzadeh S., Yıldız Z., Yildiz P., Strachowski P., Forough M., Esmaeili Y., Naebe M., Abdollahi M. (2024). Advanced macromolecular technologies in biodegradable packaging using intelligent sensing to fight food waste: A review. Int. J. Biol. Macromol..

[389] Kim K., Moon D., An J., Park S., Seo S., Ha S., Kim J., Kim K., Phyo S., Lee J. (2022). Wireless portable bioelectronic nose device for multiplex monitoring toward food freshness/spoilage. Biosens. Bioelectron..

[390] Anisimov D., Abramov A., Gaidarzhi V., Kaplun D., Agina E., Ponomarenko S. (2023). Food freshness measurements and product distinguishing by a portable electronic nose based on organic field-effect transistors. ACS Omega.

[391] Damdam A., Ozay L., Ozcan C., Alzahrani A., Helabi R., Salama K. (2023). IoT-enabled electronic nose system for beef quality monitoring and spoilage detection. Foods.

[392] Biswas B., Sahu P., Talukdar F., Baghel G. (2025). Sniffing out freshness: Gas sensors for food spoilage detection: A review. IEEE Sens. J..

[393] Mahmudiono T., Bokov D., Jasim A., Kamal W., Khashirbaeva D. (2022). State-of-the-art of convenient and low-cost electrochemical sensor for food contamination detection: Technical and analytical overview. Microchem. J..

[394] Li S., Du D., Wang J., Wei Z. (2022). Application progress of intelligent flavor sensing systems in the production process of fermented foods based on flavor properties. Crit. Rev. Food Sci. Nutr..

[395] Bodkhe G., Kumar V., Li X., Pei S., Kim M. (2025). Biosensors in microbial ecology: Revolutionizing food safety and quality. Microorganisms.

[396] Cvek M., Paul U., Zia J., Mancini G., Sedlařík V., Athanassiou A. (2022). Biodegradable films of PLA/PPC and curcumin as packaging materials and smart indicators of food spoilage. ACS Appl. Mater. Interfaces.

[397] Han Y., Wang S., Cao Y., Singh G., Loh S., Cheerlavancha R., Ang M., Khong D., Chua P., Ho P. (2023). Design of biodegradable, climate-specific packaging materials that sense food spoilage and extend shelf life. ACS Nano.

[398] Popoola O., Finny A., Dong I., Andreescu S. (2024). Smart and sustainable 3D-printed nanocellulose-based sensors for food freshness monitoring. ACS Appl. Mater. Interfaces.

[399] Singh A., Singh N., Kaur N. (2024). Design, Synthesis, and Antimicrobial Activity of Biodegradable Sodium Alginate/COF Polymeric Films for Smart Monitoring of Food Spoilage and Active Food Packaging. ACS Food Sci. Technol..

[400] Tohamy H.-A.S. (2025). Amylopectin Xerogel with Onion Based Sulfur Nitrogen Doped Carbon Quantum Dots as a Chemosensor for Chromium and Biosensor for Microbial Spoilage in Tomatoes. Sci. Rep..

[401] Jung Y., Min J., Choi J., Bang J., Jeong S., Pyun K., Ahn J., Cho Y., Hong S., Hong S. (2022). Smart paper electronics by laser-induced graphene for biodegradable real-time food spoilage monitoring. Appl. Mater. Today.

